# Adult *islet1* Expression Outlines Ventralized Derivatives Along Zebrafish Neuraxis

**DOI:** 10.3389/fnana.2019.00019

**Published:** 2019-02-26

**Authors:** Stephan W. Baeuml, Daniela Biechl, Mario F. Wullimann

**Affiliations:** Division of Neurobiology, Department Biology II, Ludwig-Maximilians-Universität München, Munich, Germany

**Keywords:** acetylcholine, alar plate, basal plate, choline acetyltransferase, dopamine, floor plate, motor nuclei, *sonic hedgehog*

## Abstract

Signals issued by dorsal roof and ventral floor plates, respectively, underlie the major patterning process of dorsalization and ventralization during vertebrate neural tube development. The ventrally produced morphogen Sonic hedgehog (SHH) is crucial for vertebrate hindbrain and spinal motor neuron development. One diagnostic gene for motor neurons is the LIM/homeodomain gene *islet1*, which has additional ventral expression domains extending into mid- and forebrain. In order to corroborate motor neuron development and, in particular, to improve on the identification of poorly documented zebrafish forebrain *islet1* populations, we studied adult brains of transgenic *islet1*-GFP zebrafish (3 and 6 months). This molecular neuroanatomical analysis was supported by immunostaining these brains for tyrosine hydroxylase (TH) or choline acetyltransferase (ChAT), respectively, revealing zebrafish catecholaminergic and cholinergic neurons. The present analysis of ChAT and *islet1*-GFP label confirms ongoing adult expression of *islet1* in zebrafish (basal plate) midbrain, hindbrain, and spinal motor neurons. In contrast, non-motor cholinergic systems lack *islet1* expression. Additional presumed basal plate *islet1* positive systems are described in detail, aided by TH staining which is particularly informative in the diencephalon. Finally, alar plate zebrafish forebrain systems with *islet1* expression are described (i.e., thalamus, preoptic region, and subpallium). We conclude that adult zebrafish continue to express *islet1* in the same brain systems as in the larva. Further, pending functional confirmation we hypothesize that the larval expression of *sonic hedgehog* (*shh*) might causally underlie much of adult *islet1* expression because it explains findings beyond ventrally located systems, for example regarding *shh* expression in the zona limitans intrathalamica and correlated *islet1*-GFP expression in the thalamus.

## Introduction

A major patterning process in the developing vertebrate neural tube (i.e., central nervous system, CNS) is the generation of gradients of morphogens both from dorsally (roof plate) and ventrally (floor plate and prechordal plate) which eventually results in dorsalization and ventralization of neural cell fates (Martí and Bovolenta, 2002; Gilbert, 2014). This process leads to differentiation of various distinct neuronal types, best known in the spinal cord and hindbrain, depending on opposed gradients of morphogens emitted by these dorsal or ventral sources. As a result, various motor neurons and related interneurons are generated ventrally, whereas sensory-related neurons (e.g., those receiving input from dorsal root ganglia) are formed dorsally (reviewed in Briscoe and Novitch, 2008; Dessaud et al., 2008; Briscoe, 2009; Grossmann et al., 2010). Critical for the ventro-dorsal signaling is the morphogen Sonic hedgehog (SHH), the activity of which has been well studied. The *shh* signaling pathway acts through the binding of SHH to the transmembraneous patched receptor, thereby freeing the default-state inhibited intracellular receptor smoothened to act on Gli activators (Briscoe and Novitch, 2008; Dessaud et al., 2008; Briscoe, 2009). Over prolonged time, the cross-repressive interactions of class I genes (repressed by SHH) and class II genes (activated by SHH) lead to differential gene expression for neuron identity in the ventral versus dorsal neural tube, including the ventral expression of *islet1*.

An early study in chick emphasized that not only spinal cord and hindbrain/midbrain floor plate, but more rostrally an even more extended *shh* expression exists in the hypothalamic basal plate and furthermore in the basal part of preoptic and telencephalic alar plate (Ericson et al., 1995) which is of utmost importance for the development of the amniote hypothalamus, preoptic region and basal ganglia. This study also showed that SHH induces the expression of follow-up genes coding for transcription factors such as the LIM/homeodomain gene *islet1* and that *islet1* is not restrictively induced in motor neurons of spinal cord and hindbrain, but also in non-motor neurons of the forebrain and, thus, that SHH is active along the entire vertebrate neuraxis (see also below the effect of SHH on telencephalic pallial *Emx1* expression in mice). However, factors additional to SHH might be involved in the telencephalon in promoting *islet1* expression.

The *sonic hedgehog* signaling pathway is also acting in zebrafish (Korzh et al., 1993; Appel et al., 1995; Tokumoto et al., 1995; Thor et al., 1999; Segawa et al., 2001; Hutchinson and Eisen, 2006; Seredick et al., 2012; Moreno and Ribera, 2014). There are three hedgehog gene groups, i.e., Sonic, Indian/Echidna and Desert hedgehog genes, seen in all vertebrate groups, each with differing expression patterns and developmental roles (Zardoya et al., 1996a,b; Avaron et al., 2006). A teleost-specific duplication furthermore led to *sonic hedgehog* (*shh*) and *tiggywinkle hedgehog* (Zardoya et al., 1996a,b). The development of amniote spinal and rhombencephalic motor neurons depends on SHH. Accordingly, mice mutant for *shh* show no dorsoventral patterning in the spinal cord as exemplified with diagnostic *Pax 2,4* and *6* gene expression (Chiang et al., 1996). Furthermore, such mice lack motor neurons and show no *islet1* expression (Litingtung and Chiang, 2000). Moreover, mice mutant for *shh* show an extension of the pallialy expressed gene *Emx1* into the basal telencephalon (Chiang et al., 1996). While the knockout of mammalian *shh* is sufficient for these effects (Chiang et al., 1996; Litingtung and Chiang, 2000), in zebrafish, only the knockout of three hedgehog genes (*sonic hedgehog*, *echidna*, and *tiggywinkle hedgehog*) is sufficient for the loss of motor neurons (and *islet1* expression), whereby *echidna* seems the least important of the three (Eisen, 1999; Lewis and Eisen, 2001). In line with this, zebrafish expression domains of *shh* and *tiggywinkle hedgehog* include prechordal/notochordal mesoderm, floor plate and ventral forebrain, while that of *echidna hedgehog* is in later notochord only (Lewis and Eisen, 2001).

These previous studies in zebrafish primarily focused on early differentiation of spinal cord and hindbrain giving little regard to forebrain. In order to fill in this gap, we here look in great detail at *islet1* expression in the differentiated adult zebrafish brain (3 months, with some additional information at 6 months). At the same time, adult *islet1* expression in the posterior brain will be revealed. To this aim, we used a transgenic zebrafish line which shows specifically *islet1*-GFP expression in cranial nerve motor neurons, but also in forebrain neurons (Higashijima et al., 2000). However, our report shows in far greater detail the adult expression patterns in this *islet1*-GFP line. An additional fortuitous point in studying this transgenic line is that due to the cytoplasmic localization of GFP, fibers are visualized also, allowing for gaining information on main tracts issued by *islet1*-GFP neurons.

By uncovering *islet1* expressing structures in the adult central nervous system, we propose that we delineate a fraction of CNS systems which likely depend on early *shh* activity. This is a working hypothesis because we do not provide data to show that all these *islet1* expressing systems mechanistically depend on upstream *shh* expression. Also, there are surely additional *shh* depending (non-*islet1* expressing systems). In addition, we summarize from our previous data pool the larval *shh* expression and discuss the possible developmental implications for each brain part.

Our laboratory (Rink and Wullimann, 2001; Mueller et al., 2004; Yamamoto et al., 2011; Wullimann, 2014) and others (Ma, 1994a,b, 1997, 2003; Kaslin and Panula, 2001; Clemente et al., 2004; Kaslin et al., 2004; Castro et al., 2006a) previously provided complete descriptions and identifications of catecholaminergic and cholinergic systems in the adult zebrafish brain. In the present contribution, we additionally counterstain *islet1*-GFP adult zebrafish brain sections with antibodies either against tyrosine hydroxylase (TH) or choline acetyltransferase (ChAT). The latter will visualize motor neurons from midbrain to spinal cord and the former all catecholamine systems (e.g., complicated diencephalic dopamine systems) and clarify in detail which systems are *islet1*-GFP positive. This parallel demonstration of these modulatory systems greatly helps in the neuroanatomical analysis, which was primarily based on an overall histological nuclear stain (DAPI).

Thus, the present contribution shows that there is long-lasting, continued expression of *islet1* in the adult zebrafish brain and, because of the advanced degree of brain differentiation, provides a map of those ventralized systems depending on the *shh-islet1* pathway.

## Materials and Methods

### Transgenic Zebrafish Strains

The transgenic line Tg(2.4*shh*a-*ABC-GFP*)sb15 was originally published as Tg(*2.2shh:gfpABC#15*) by Shkumatava et al. (2004) and will be referred to in the following as *shh-*GFP line. It has been already used before by our lab to study larval expression of *shh*-GFP (Biechl et al., 2016). Details for the generation of these specimens, as well as origin of brain sections depicted in this contribution, are given in this previous paper.

The transgenic *islet1*-GFP line Tg(*isl1:GFP*) used in this contribution was generated specifically to show expression of GFP in cranial nerve motor neurons (Higashijima et al., 2000). We raised zebrafish *islet1-*GFP specimens into larval stages and up to 3 and 6 months. Fish were maintained according to standard protocols (Westerfield, 2007).

All procedures involving live zebrafish were carried out according to EU guidelines and German legislation (EU Directive 2010_63, license number AZ 325.1.53/56.1-TU-BS). Transgenic animals used in this study were killed with an overdose of tricaine methane sulfonate (MS-222) and fixed in paraformaldehyde (4% PFA in Sörensen’s phosphate buffer, PB) at 4°C overnight. The raising and fixation of these transgenic animals was performed in Prof. Reinhard Köster’s lab (Technical University Braunschweig, Germany) and kindly subsequently provided to us. Therefore, our study only involved fixed animal tissue and needed no further approval.

### Cutting Procedure

Following cryoprotection in sucrose solution (30% sucrose solution at 4°C overnight), adult brains were embedded in TissueTek (tissue freezing medium, A. Hartenstein GmbH) and cryosectioned (Leica, CM 3050 S) at 30 μm before thaw mounted onto Superfrost Plus glass slides (Thermo) and coverslipped after immunoprocedures. Totally, eight 3-month old and two 6-month old specimens were used in this study.

### Immunohistochemical Processing

Notably, transgenic animal tissue was kept as far as possible in darkness during histological processing due to the photosensitivity of the GFP-protein. Immunohistochemical incubations were done in a humid chamber. After washing off TissueTek in cryosections with phosphate buffered saline (PBS), endogenous peroxidase activity was first blocked with 0.3% H_2_O_2_ in PBS for 30 min at room temperature (RT), washed in PBT (PBS + 0.1% Tween20) and blocked with blocking buffer (2% normal goat serum, 2% bovine serum albumin, 0.2% Tween20, 0.2% TritonX- 100 in PBS) for 1 h at RT before exposition to a primary antibody against GFP diluted in blocking buffer at 4°C for 1–3 days (dilutions see Table 1). After washing in PBT, the sections were incubated with the secondary antibody (see Table 1) diluted in blocking solution overnight at 4°C. Subsequently, a second primary antibody (against TH or ChAT, see Table 1) was applied after intermittent washing in PBT and blocking (see above for details), followed by the application of the appropriate secondary antibody (see Table 1) diluted in blocking buffer overnight, after intermittent washing in PBT and blocking (see above for details). Finally, sections were washed in PBT and counterstained with DAPI (40-6-diamidino-2-phenylindole; Carl Roth, 1:1000) and washed in PBS. Slides were then mounted with Vectashield (Vectorlabs) and coverslipped. Previously, various controls and Western blot analysis for the antibody against tyrosine hydroxylase have been performed (Yamamoto et al., 2010, 2011). Also, the ChAT antibody has been used previously (Mueller et al., 2004; see there for its characterization and specificity).

**Table 1:** Antibodies.

Antibody against	Host	Company	Dilution
TH	Mouse, monoclonal	Millipore (AbCys), #MAB318	1:100
2nd	Donkey (Anti-mouse-Cy3)	Dianova, #715-166-151	1:400
ChAT	Goat, polyclonal	Millipore (Chemicon), #AB144P-200 UL	1:100
2nd	Donkey (Anti-goat-Cy3)	Dianova #705-165-147	1:400
GFP	Chicken	Aves Labs #GFP-1020	1:500
2nd	Donkey (Anti-chicken-FITC)	Dianova (Mol. Probes) #A11039 703-095155	1:100


We furthermore checked for differences between the intrinsic GFP signal with the one enhanced through use of the anti-GFP antibody and found no neuroanatomical differences.

### Photography

Cryostat sections of adult zebrafish heads were photographed using a light/fluorescence microscope (Nikon Eclipse 80i; Nikon Instruments Inc.) with a Nikon Digital Sight DSU1 Photomicrographic Camera (Nikon Instruments Inc.) and LUCIA-G5 software or NIS-Elements F4.60.00 software. The microscope was equipped with Nikon Plan UW 0.06 (2×), Plan Fluor 109/0.30 (10×) and Plan Fluor 209/.0.50 (20×) objectives.

All images were eventually slightly adapted for brightness and contrast with Corel PHOTO-PAINT 9.0 and mounted into figures with Corel DRAW 9.0 (Corel Corporation, Ottawa, ON, Canada).

### Analysis of Data

**FIGURE 1:**
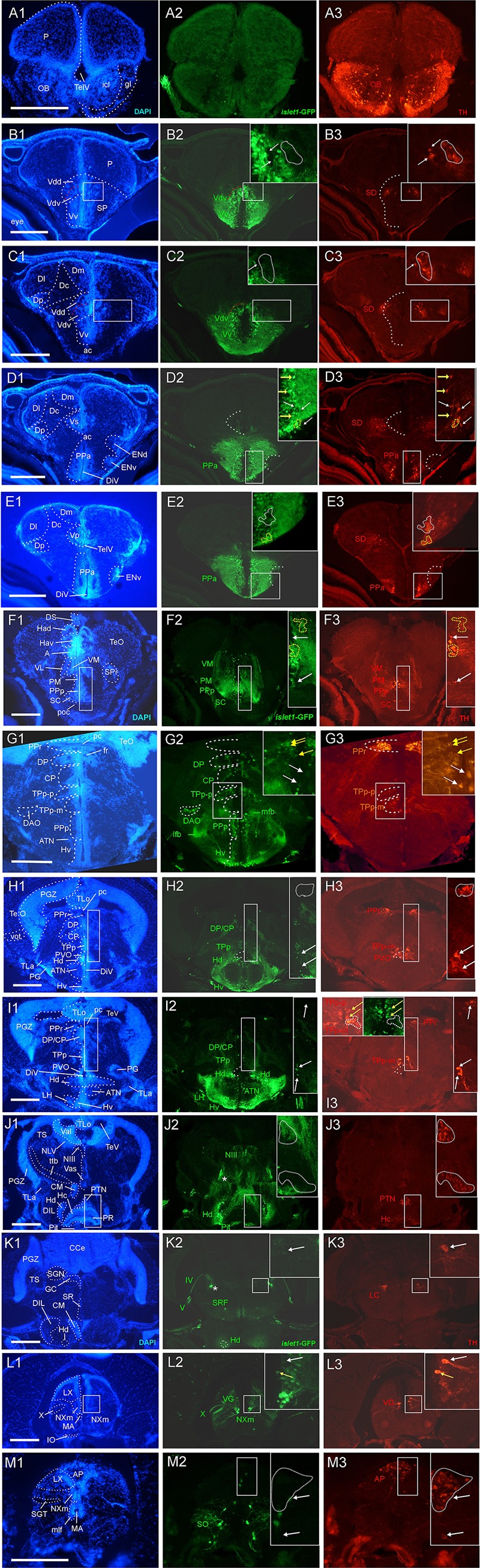
Analysis of *islet1*-GFP expression in the adult zebrafish brain using a rostrocaudal series of sections showing also fluorescent DAPI stain and tyrosine hydroxylase (TH) immunohistochemistry from olfactory bulb **(A)** to hindbrain **(M)**. The left column **(A1–M1)** presents a general neuroanatomical analysis performed on the nuclear stain DAPI. In the middle column **(A2–M2)**, only brain nuclei containing *islet1*-GFP cell bodies and motor cranial nerves, but not other stained fibers, are identified in green lettering. In the right column **(A3–M3)** only brain nuclei containing TH positive cell bodies are identified in red lettering. White arrows/fine stippled lines point to/encircle single labeled cell bodies/cell populations, whereas yellow arrows/fine stippled lines point to/encircle double-labeled cell bodies/cell populations. Occasionally, selected structures pointed out in DAPI stains with coarse stippled lines are also indicated in the *islet1*-GFP and TH pictures to ease identification. In **(B,C)**, the boundary between dorsal and ventral parts of the dorsal nucleus of the subpallium is indicated with a red stippled line. In **(I3)**, the inset in the upper left corner is taken from a different specimen. A, anterior thalamic nucleus; ac, anterior commissure; ALLN, anterior lateral line nerve; AP, area postrema; ATN, anterior tuberal nucleus; C, central canal; CC, crista cerebellaris; CCe, corpus cerebelli; cgus, commissure of secondary gustatory nuclei; cinf, commissura infima of Haller; CM, corpus mamillare; CON, caudal octavolateralis nucleus; CP, central posterior thalamic nucleus; cven, ventral rhombencephalic commissure; DAO, dorsal accessory optic nucleus; Dc, central zone of dorsal telencephalon; dIV, decussation of trochlear nerve; DIL, diffuse nucleus of inferior lobe; DiV, diencephalic ventricle; Dl, lateral zone of dorsal telencephalon; Dm, medial zone of dorsal telencephalon; DON, descending octaval nucleus; DP, dorsal posterior thalamic nucleus; Dp, posterior zone of dorsal telencephalon; DS, saccus dorsalis; DT, dorsal thalamus; DTN, dorsal tegmental nucleus; EG, eminentia granularis; End/ENv, dorsal/ventral entopeduncular nucleus; fr, fasciculus retroflexus; GC, griseum centrale; gl, glomerular layer (olfactory bulb); Had/Hav, dorsal/ventral habenular nucleus; Hc/Hd/Hv, caudal/dorsal/ventral zone of periventricular hypothalamus; iaf, internal arcuate fibers; icl, inner granular cell layer (olfactory bulb); IMRF, intermediate reticular formation; IN, intermediate hypothalamic nucleus; IO, inferior olive; IR, inferior raphe; IRF, inferior reticular formation; LIX, lobus glossopharyngeus; LVII, lobus facialis; LX, lobus vagus; LC, locus coeruleus; lfb, lateral forebrain bundle; LH, lateral hypothalamic nucleus; MA, Mauthner axon; mfb, medial forebrain bundle; MFN, medial funicular nucleus; mlf, medial longitudinal fascicle; MON, medial octavolateralis nucleus; NIII, oculomotor nerve nucleus; NIV, trochlear motor nerve nucleus; NIXm, glossopharyngeal motor nerve nucleus; NVmd, dorsal trigeminal motor nerve nucleus; NVmv, ventral trigeminal motor nerve nucleus; NVs, primary sensory trigeminal nucleus; NVIc/NVIr, caudal/rostral abducens motor nerve nucleus; NVIImc/NVIImr, caudal/rostral facial motor nerve nucleus; NXm, vagal motor nerve nucleus; pc, posterior commissure; NI, nucleus isthmi; NIn, Nucleus interpeduncularis; NLV, nucleus lateralis valvulae; OB, olfactory bulb; OENc/OENr, caudal/rostral octavolateralis efferent neurons; P, pallium; pc, posterior commissure; PG, preglomerular complex; PGm, medial preglomerular nucleus; PGZ, periventricular gray zone of optic tectum; Pit, pituitary; PLc, caudal perilemniscal nucleus; PM, magnocellular preoptic nucleus; poc, postoptic commissure; PPa/PPp, anterior/posterior parvocellular preoptic nucleus; PPr, periventricular pretectum; PR, posterior hypothalamic recess; PTN, posterior tuberal nucleus; PVO, paraventricular organ; RT, rostral tegmental nucleus (of Grover and Sharma, 1981); RV, rhombencephalic ventricle; SC, suprachiasmatic nucleus; SD, subpallial dopaminergic cells; SGN, secondary gustatory nucleus; SGT, secondary gustatory tract; SP, subpallium; SPr, superficial pretectum; SO, spino-occipital region; SR, superior raphe; SRF, superior reticular formation; SRN, superior reticular nucleus; TelV, telencephalic ventricle; TeO, optic tectum; TeV, tectal ventricle; TLa, torus lateralis; TLo, torus longitudinalis; TPp, periventricular nucleus of posterior tuberculum; TPp-p, parvocellular cell part of TPp; TPp-m, magnocellular (pear-shaped) cell part of TPp; TS, torus semicircularis; ttb, tractus tecto-bulbaris; Val, lateral part of valvula cerebelli; Vam, medial part of valvula cerebelli; Vas, vascular lacuna; Vdd/Vdv, dorsal/ventral subnucleus of dorsal nucleus of ventral telencephalon; VG, vagal group of catecholamine neurons; VH, ventral horn; VL/VM, ventrolatateral/ventromedial thalamic nucleus; vot, ventrolateral optic tract; Vp/Vs/Vv, posterior/supracommissural/ventral nucleus of ventral telencephalon; vr, ventral spinal root; Vsr, sensory trigeminal root; IV/V/VIII/IX/X, trochlear, trigeminal, octaval, glossopharyngeal, vagal nerve (motor components).

**FIGURE 2:**
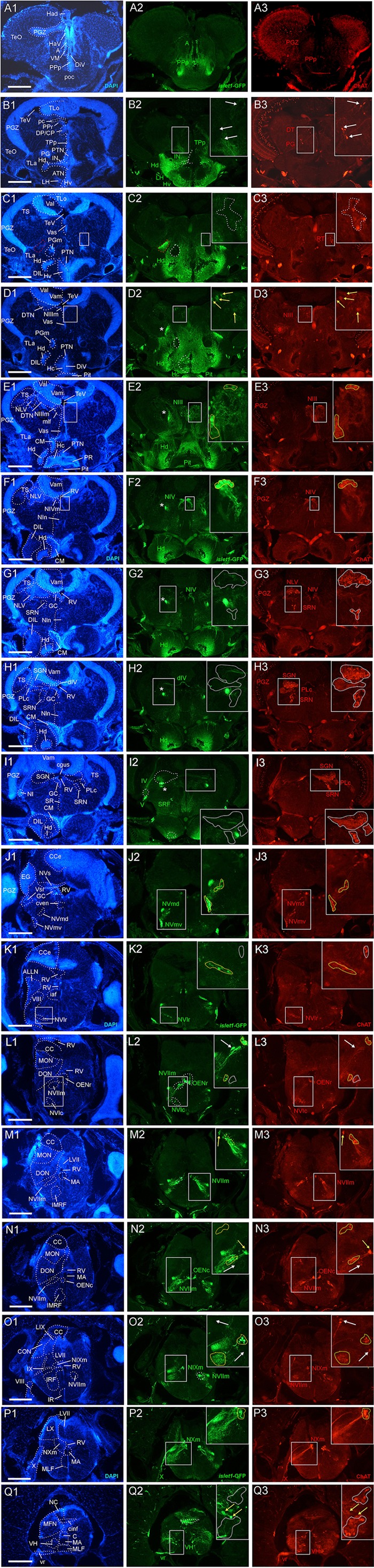
Analysis of *islet1*-GFP expression in the adult zebrafish brain using a rostrocaudal series of sections showing also fluorescent DAPI stain and choline acetyltransferase (ChAT) immunohistochemistry from olfactory bulb **(A)** to spinal cord **(Q)**. The left column **(A1–Q1)** presents a general neuroanatomical analysis performed on the nuclear stain DAPI. In the middle column **(A2–Q2)**, only brain nuclei containing *islet1*-GFP cell bodies and motor cranial nerves, but not other stained fibers, are identified in green lettering. In the right column **(A3–Q3)** only brain nuclei containing ChAT positive cell bodies and some motor nerve fibers are identified in red lettering. White arrows/fine stippled lines point to/encircle single labeled cell bodies/cell populations, whereas yellow arrows/fine stippled lines point to/encircle double-labeled cell bodies/cell populations. Occasionally, selected structures pointed out in DAPI stains with coarse stippled lines are also indicated in the *islet1*-GFP and ChAT pictures to ease identification. A conspicuous *islet1*-GFP positive tract is present from the level of the medial preglomerular nucleus where it is encircled with a red coarse stippled line **(C1,C2)** and then identified with an asterisk into the isthmic level **(D2–I2)**. #: indicates in **(D3–H3)** a ChAT positive fiber net (see Mueller et al., 2004). A, anterior thalamic nucleus; ac, anterior commissure; ALLN, anterior lateral line nerve; AP, area postrema; ATN, anterior tuberal nucleus; C, central canal; CC, crista cerebellaris; CCe, corpus cerebelli; cgus, commissure of secondary gustatory nuclei; cinf, commissura infima of Haller; CM, corpus mamillare; CON, caudal octavolateralis nucleus; CP, central posterior thalamic nucleus; cven, ventral rhombencephalic commissure; DAO, dorsal accessory optic nucleus; Dc, central zone of dorsal telencephalon; dIV, decussation of trochlear nerve; DIL, diffuse nucleus of inferior lobe; DiV, diencephalic ventricle; Dl, lateral zone of dorsal telencephalon; Dm, medial zone of dorsal telencephalon; DON, descending octaval nucleus; DP, dorsal posterior thalamic nucleus; Dp, posterior zone of dorsal telencephalon; DS, saccus dorsalis; DT, dorsal thalamus; DTN, dorsal tegmental nucleus; EG, eminentia granularis; End/ENv, dorsal/ventral entopeduncular nucleus; fr, fasciculus retroflexus; GC, griseum centrale; gl, glomerular layer (olfactory bulb); Had/Hav, dorsal/ventral habenular nucleus; Hc/Hd/Hv, caudal/dorsal/ventral zone of periventricular hypothalamus; iaf, internal arcuate fibers; icl, inner granular cell layer (olfactory bulb); IMRF, intermediate reticular formation; IN, intermediate hypothalamic nucleus; IO, inferior olive; IR, inferior raphe; IRF, inferior reticular formation; LIX, lobus glossopharyngeus; LVII, lobus facialis; LX, lobus vagus; LC, locus coeruleus; lfb, lateral forebrain bundle; LH, lateral hypothalamic nucleus; MA, Mauthner axon; mfb, medial forebrain bundle; MFN, medial funicular nucleus; mlf, medial longitudinal fascicle; MON, medial octavolateralis nucleus; NIII, oculomotor nerve nucleus; NIV, trochlear motor nerve nucleus; NIXm, glossopharyngeal motor nerve nucleus; NVmd, dorsal trigeminal motor nerve nucleus; NVmv, ventral trigeminal motor nerve nucleus; NVs, primary sensory trigeminal nucleus; NVIc/NVIr, caudal/rostral abducens motor nerve nucleus; NVIImc/NVIImr, caudal/rostral facial motor nerve nucleus; NXm, vagal motor nerve nucleus; pc, posterior commissure; NI, nucleus isthmi; NIn, Nucleus interpeduncularis; NLV, nucleus lateralis valvulae; OB, olfactory bulb; OENc/OENr, caudal/rostral octavolateralis efferent neurons; P, pallium; pc, posterior commissure; PG, preglomerular complex; PGm, medial preglomerular nucleus; PGZ, periventricular gray zone of optic tectum; Pit, pituitary; PLc, caudal perilemniscal nucleus; PM, magnocellular preoptic nucleus; poc, postoptic commissure; PPa/PPp, anterior/posterior parvocellular preoptic nucleus; PPr, periventricular pretectum; PR, posterior hypothalamic recess; PTN, posterior tuberal nucleus; PVO, paraventricular organ; RT, rostral tegmental nucleus (of Grover and Sharma, 1981); RV, rhombencephalic ventricle; SC, suprachiasmatic nucleus; SD, subpallial dopaminergic cells; SGN, secondary gustatory nucleus; SGT, secondary gustatory tract; SP, subpallium; SPr, superficial pretectum; SO, spino-occipital region; SR, superior raphe; SRF, superior reticular formation; SRN, superior reticular nucleus; TelV, telencephalic ventricle; TeO, optic tectum; TeV, tectal ventricle; TLa, torus lateralis; TLo, torus longitudinalis; TPp, periventricular nucleus of posterior tuberculum; TPp-p, parvocellular cell part of TPp; TPp-m, magnocellular (pear-shaped) cell part of TPp; TS, torus semicircularis; ttb, tractus tecto-bulbaris; Val, lateral part of valvula cerebelli; Vam, medial part of valvula cerebelli; Vas, vascular lacuna; Vdd/Vdv, dorsal/ventral subnucleus of dorsal nucleus of ventral telencephalon; VG, vagal group of catecholamine neurons; VH, ventral horn; VL/VM, ventrolatateral/ventromedial thalamic nucleus; vot, ventrolateral optic tract; Vp/Vs/Vv, posterior/supracommissural/ventral nucleus of ventral telencephalon; vr, ventral spinal root; Vsr, sensory trigeminal root; IV/V/VIII/IX/X, trochlear, trigeminal, octaval, glossopharyngeal, vagal nerve (motor components).

Each section shown in Figure 1A–M was photographed in three appropriate fluorescent spectral channels for the presence of the nuclear stain DAPI, *islet1*-GFP, and tyrosine hydroxylase (TH) or, in Figure 2A–Q, alternatively for the presence of choline acetyltransferase (ChAT) instead of TH. Subsequently, the ImageJ tool of synchronizing all windows was used to analyze cellular co-localization of *islet1*-GFP with either TH or ChAT on a neuroanatomical background yielded by the DAPI pictures. Since the three microphotographs were identical in each case except for the fluorescence visualized, we could assign in detail to a cell nucleus seen in DAPI stain the associated cytoplasmic green GFP and red transmitter-related enzyme stain on a cell to cell basis.

In many cases, critical regions of interest are identified with rectangles in the figures and are shown as enlarged insets where double-label for TH/*islet1*-GFP or ChAT/*islet1*-GFP is indicated with yellow arrows or surrounded by yellow stippled lines. In contrast, white arrows/white stippled lines indicate single-labeled structures.

## Results

We will first give a detailed account on *islet1*-GFP expression in the 3 months adult zebrafish brain. Then we will analyze which catecholaminergic and which cholinergic structures are co-localized with *islet1*-GFP. Finally, we summarize larval *sonic hedgehog* expression using a *shh*-GFP line (previously investigated in more detail, see Biechl et al., 2016). The *shh* expression domains are informative for the explanation of *islet1* expression patterns because of the former’s role as an important upstream gene of *islet1* (see section “Introduction”).

For identification of brain structures, basically the adult *Neuroanatomy of the Zebrafish* (Wullimann et al., 1996) was used with the following five important updates that have been made since.

The zona limitans (note that there is no relationship to the embryonic zona limitans intrathalamica mentioned below) between posterior tuberculum and hypothalamus in said atlas (Wullimann et al., 1996, p. 36) is included in the paraventricular organ here. Further, the paraventricular organ of the atlas (Wullimann et al., 1996, p. 39) is now the intermediate hypothalamic nucleus. These two changes were justified in Rink and Wullimann (2001) based on new data of TH expression. Thirdly, the cholinergic superior reticular nucleus was misidentified in the original atlas as the rostral part of the dorsal motor trigeminal nucleus (see Mueller et al., 2004).

The periventricular zones of the hypothalamus include the ventral zone (Hv; anterior and ventral to the lateral recess), the dorsal zone (Hd; around the lateral recess, including the intermediate hypothalamic nucleus, see above) and the caudal zone (Hc; posterior to the lateral recess). The Hc includes an unpaired anterior midline portion and a posterior part that expands bilaterally around the emerging posterior recess. Both anterior and posterior parts of the Hc are characterized by TH positive cells (Rink and Wullimann, 2001; Yamamoto et al., 2010, 2011), whereas the ventral and dorsal hypothalamic zones contain no or a few TH positive cells, respectively. This was confirmed by strong expression of the *TH1* and particularly of the *TH2* gene in both parts of Hc. Because the TH2 enzyme is at times poorly visualized with TH immunohistochemistry (IHC), it first came as a surprise that the (TH negative) intermediate hypothalamic nucleus (embedded in Hd) expresses strongly *TH2* and also synthesizes dopamine (Yamamoto et al., 2010, 2011). In contrast, the TH positive posterior tuberal nucleus (PTN) lying in the midline dorsal to the caudal hypothalamus expresses only *TH1* (but not *TH2*). The PTN is prominently seen with TH-IHC and can be identified dorsally down to the most posterior Hc (Rink and Wullimann, 2001; Yamamoto et al., 2010, 2011, present contribution). This is the fourth deviation from the atlas where this most caudal part of PTN had been included in Hc (Wullimann et al., 1996, p. 41).

Finally, we identify the area postrema in line with previous research in zebrafish and other teleosts (Hornby et al., 1987; Morita and Finger, 1987; Hornby and Piekut, 1988; Manso et al., 1993; Ma, 1997; Kaslin and Panula, 2001; Castro et al., 2006b) here as the catecholaminergic dorsal population in the very posterior caudal hindbrain that lies dorsally between vagal lobes and the commissural nuclei of Cajal (see justification in Kress and Wullimann, 2012) and no longer as associated with the vascular lacunae seen in the area of the nucleus of the medial longitudinal fascicle (Wullimann et al., 1996, p. 42).

### *islet1*-GFP Expression

The transgenic zebrafish *islet1*-GFP line beautifully maintains qualitatively all brain expression sites into adulthood which were partly in detail (for example in the hindbrain) and partly more globally (in particular in the forebrain) already known from embryonic or larval stages (see section “Discussion”). Thus, with due cautiousness, we propose that this comparison shows that no qualitative changes between larval and adult *islet1*-GFP brain expression exist. In any case, our adult brain expression analysis allows for a detailed neuroanatomical allocation of *islet1* expressing structures because of the zebrafish brain’s progressed differentiation state. For the following analysis we will use neuroanatomical structures as visualized by the fluorescent nuclear stain DAPI (left vertical column in Figure 1, 2) and compare them with *islet1*-GFP expression in the same zebrafish brain sections (middle vertical column in Figure 1, 2). A complete list of *islet1*-GFP positive brain nuclei can be gathered from Tables 2–4.

**Table 2:** Catecholaminergic brain nuclei and *islet1*-GFP.

Structure	TH	*islet1*-GFP	Co-localization
Olfactory bulb (OB)	+	-	-
Subpallial dopamine cells (SD) associated with ventral telencephalic nuclei (Vd, Vv, Vs)	+	-	-
Anterior parvocellular preoptic nucleus (PPa)	+	+	+
Posterior parvocellular preoptic nucleus (PPp)	+	+	+
Magnocellular preoptic nucleus (PM)	(+)	(+)	-
Suprachiasmatic nucleus (SC)	(+)	(+)	-
Ventral thalamus (VT, ∼Zona incerta)	+	+	+
Periventricular pretectal nucleus (PPr)	+	-	-
Small cells of periventricular posterior tubercular nucleus (TPp-p)	+	+	+
Large cells of posterior tubercular nucleus (TPp-m)	+	-	-
Paraventricular organ (PVO)	+	-	-
Posterior tuberal nucleus (PTN)	+	-	-
Posterior part of caudal zone of periventricular hypothalamus (Hc)	+	-	-
Locus coeruleus (LC)	+	-	-
Vagal group of catecholaminergic neurons (VG)	(+)	(+)	(+)
Area postrema (AP)	+	-	-


*(+) few cells.*

**Table 3:** Cholinergic brain nuclei and *islet1*-GFP.

Structure	ChAT	*islet1*-GFP	Co-localization
Lateral nucleus of ventral telencephalon (Vl)	(+)^∗^	-	-
Anterior parvocellular preoptic nucleus (PPa)	(+)	+	-
Posterior parvocellular preoptic nucleus (PPp)	(+)	+	-
Magnocellular preoptic nucleus (PM)	(+)	(+)	-
Suprachiasmatic nucleus (SC)	(+)	(+)	-
Dorsal thalamus (DT)	(+)	(+)	-
Cells near preglomerular complex (PG)^∗∗^	(+)	-	-
Rostral tegmental nucleus (RT)	+	-	-
Periventricular gray zone of optic tectum (SGZ)	+	-	-
Oculomotor nerve nucleus (NIII)	+	+	+
Edinger–Westphal nucleus (NEW)	+	+	+
Hindbrain motor nerve nuclei IV–VII, IX, X	+	+	+
Octavolateralis efferent neurons (e)	+	+	+
Superior reticular nucleus (SRN)	+	-	-
Nucleus lateralis valvulae (NLV)	+	-	-
Nucleus isthmi (NI)	+	-	-
Secondary gustatory nucleus (SGN)	+	-	-
Caudal perilemniscal nucleus (PLc)	+	-	-
Cells ventrolateral to caudal perilemniscal nucleus	+	+	+


**Table 4:** *islet1*-GFP positive brain nuclei containing neither TH nor ChAT.

Structure	TH/ChAT	*islet1*-GFP	Co-localization
Ventral, dorsal and supracommissural ventral telencephalic nuclei (Vv, Vd, Vs)	-	+	-
Dorsal accessory optic nucleus (DAO)	-	+	-
Nucleus of the medial longitudinal fascicle (Nmlf)	-	+	-
Anterior hypothalamic nucleus (ATN)	-	+	-
Dorsal zone of periventricular hypothalamus (Hd)	-	+	-
Lateral hypothalamic nucleus (LH)	-	+	-
Intermediate hypothalamic nucleus (IN)	-^∗^	+	-
Ventral zone of periventricular hypothalamus (Hv)	-	+	-
Anterior part of caudal zone of periventricular hypothalamus (Hc)	-^∗^	+	-
Pituitary (Pit)	-	+	-
Rostral perilemniscal nucleus (PLr)	-	+	-
Superior reticular formation (SRF)	-	+	-
Spino-occipital region (SO)		+	-


*^∗^Note that these cells express *TH2* – not visualized with TH antibodies – and contain dopamine (Yamamoto et al., 2011) and may thus potentially be double-labeled with *islet1*-GFP.*

**FIGURE 3:**
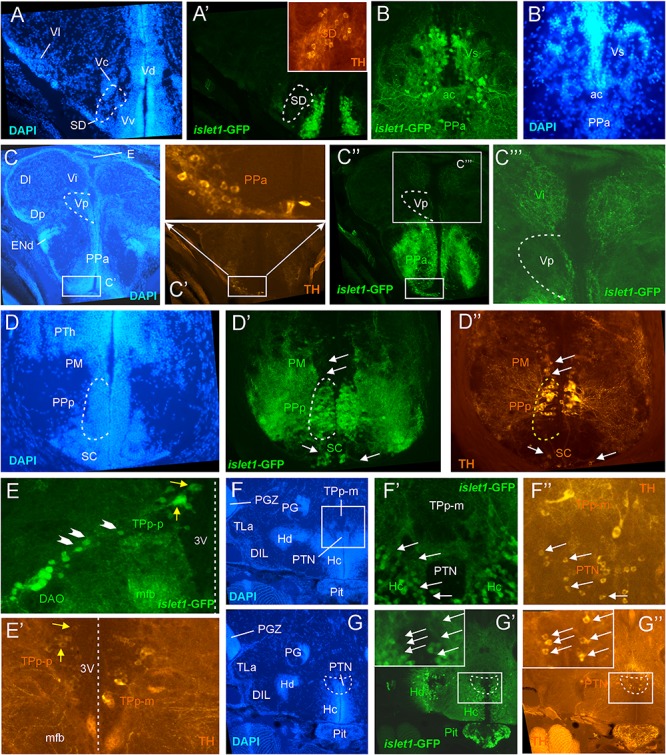
Details of *islet1*-GFP expression using fluorescent DAPI stain and tyrosine hydroxylase (TH) immunohistochemistry. Conventions with lettering as in Figure 1. **(A,A’)** *islet1* expression in precommissural subpallium shows positivity in ventral, and negativity in central (Vc) and lateral nuclei (Vl). **(B,B’)** *islet1*-GFP expression in supracommissural nucleus (Vs). **(C–C”’)** *islet1*-GFP fibers in intermediate nucleus of the ventral telencephalon. Note also TH cells in the anterior parvocellular preoptic nucleus (PPa). **(D–D”)** Dopamine cells in magnocellular (PM), posterior parvocellular (PPp), and suprachiasmatic nuclei (SC), with only cells in PPp double-labeled for *islet1*-GFP. **(E)** Origin of the dorsal accessory optic nucleus (DAO) as suggested by a chain of *islet1*-GFP cells (arrowheads) that apparently migrate pially from the parvocellular periventricular posterior tubercular nucleus (TPp-p) and merge into the DAO. Straight stippled line indicates midline. **(E’)** Slightly more medially taken picture shows the parvocellular (TPp-p) and pear-shaped (TPp-m) parts of TPp on both brain sides in TH immunostaining. Note that some cells in TPp-p are double-labeled. **(F–G)** Analysis of anterior part of caudal zone of periventricular hypothalamus (Hc) and posterior tuberal nucleus (PTN) at rostral (**F**: DAPI, **F’**: *islet1*-GFP, **F”**: TH stain) and caudal levels (**G**: DAPI, **G’**: islet1-GFP, **G”**: TH stain). Note that there are no TH and *islet1*-GF double-labeled cells in PTN. ac, anterior commissure; DAO, dorsal accessory optic nucleus; DIL, diffuse nucleus of the inferior lobe; Dl, lateral zone of dorsal telencephalon; Dm, medial zone of the dorsal telencephalon; DP, dorsal posterior thalamic nucleus; E, epiphysis (pineal); ENd, dorsal entopeduncular nucleus; Hc/Hd, caudal/dorsal zone of periventricular hypothalamus; mfb, medial forebrain bundle; PG, preglomerular complex; PGZ, periventricular gray zone of optic tectum; Pit, pituitary; PM, magnocellular preoptic nucleus; PPa/PPp, anterior/posterior parvocellular preoptic nucleus; PTh, prethalamus; PTN, posterior tuberal nucleus; TLa, torus lateralis; TPp-m/TPP-p, magnocellular (pear-shaped)/parvocellular cell part of periventricular posterior tubercular nucleus; Vc/Vd/Vi/Vl/Vp/Vs/Vv, central/dorsal/intermediate/lateral/posterior/ supracommissural/ventral nucleus of ventral telencephalon; SC, suprachiasmatic nucleus; SD, subpallial dopamine cells; VI, abducens nerve.

**FIGURE 4:**
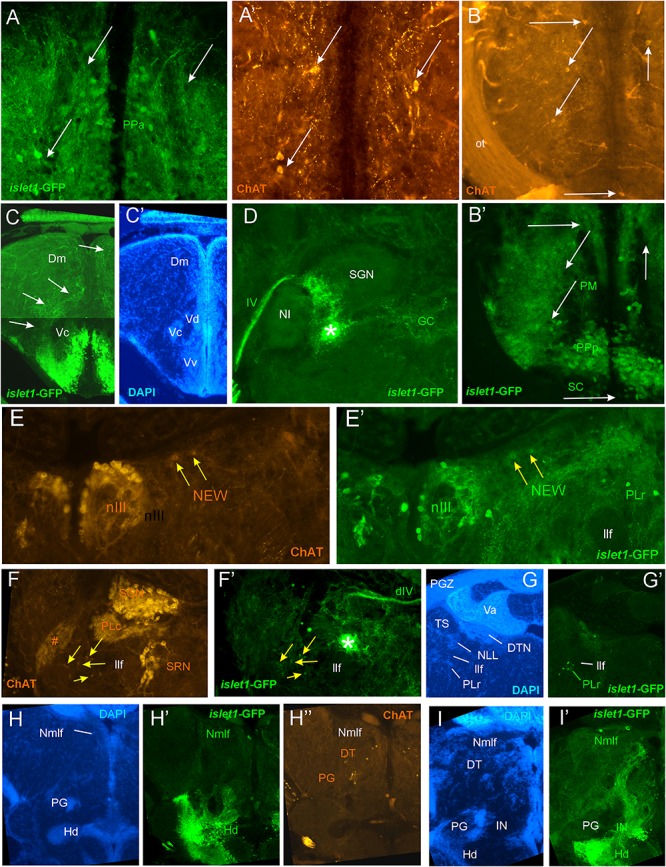
Details of *islet1*-GFP expression using fluorescent DAPI stain and choline acetyltransferase (ChAT) immunohistochemistry. Conventions with lettering as in Figure 2. **(A,A’)** Cholinergic cells in anterior parvocellular preoptic nucleus (PPa) are never double-labeled with *islet1*-GFP. **(B,B’)** Cholinergic cells in magnocellular and posterior parvocellular preoptic nuclei (PM, PPp) and suprachiasmatic nucleus (SC) are never double-labeled with *islet1*-GFP. **(C,C’)** Shows *islet1*-GFP positive axonal tract from the ventral nucleus of the ventral telencephalon (Vv) to the medial zone of the dorsal telencephalon (Dm). **(D)** Magnification shows how hypothalamic tract leads into the medial part of the secondary gustatory nucleus with some collaterals into the central gray. **(E,E’)** Magnification of midbrain tegmentum shows lateral to the oculomotor nerve nucleus (NIII) the additional cholinergic cells of the nucleus of Edinger–Westphal (NEW; yellow arrows) to be double-labeled for *islet1*-GFP. Note also the few non-cholinergic cells of the rostral perilemniscal nucleus stained for *islet1*-GFP. **(F,F’)** Highlights the few cholinergic/*islet1*-GFP positive cells (yellow arrows) ventrolateral to the caudal perilemniscal nucleus which itself is *islet1*-GFP negative, but ChAT positive. #: indicates a ChAT positive fiber net (See Mueller et al., 2004). **(G,G’)** *islet1*-GFP cells in the rostral perilemniscal nucleus (PLr). **(H–H”)** *islet1*-GFP positive cells in the nucleus of the medial longitudinal fascicle (Nmlf) and cholinergic cells in the dorsal thalamus (DT) and near the preglomerular region (PG). **(I,I’)** *islet1*-GFP cells in the Nmlf or possibly the mesencephalic sensory trigeminal nucleus at the level of an *islet1*-GFP axonal tract that projects from the intermediate hypothalamic nucleus (IN) to the dorsal thalamus. dIV, decussation of trochlear nerve; Dm, medial zone of dorsal telencephalon; DT, dorsal thalamus; DTN, dorsal tegmental nucleus; GC, griseum centrale; Hd, dorsal zone of periventricular hypothalamus; IN, intermediate hypothalamic nucleus; IIf, lateral forebrain bundle; NIII, oculomotor nerve nucleus; NEW, nucleus of Edinger–Westphal; NI, nucleus isthmi; NLL, nucleus of the lateral lemniscus (of Prasada Rao et al., 1987); Nmlf, nucleus of the medial longitudinal fascicle; ot, optic tract; PG, preglomerular complex; PGZ, periventricular layer of optic tectum; PLc/PLr, caudal/rostral perilemniscal nucleus; PM, magnocellular preoptic nucleus; PPa/PPp, anterior/posterior parvocellular preoptic nucleus; SC, suprachiasmatic nucleus; SGN, secondary gustatory nucleus; SRN, superior reticular nucleus; TS, torus semicircularis; Va, valvula cerebelli; Vc/Vd/Vv, central/dorsal/ventral nucleus of ventral telencephalon; IV, trochlear nerve.

In the telencephalon, all dorsal (pallial) divisions lack any trace of *islet1*-GFP expression in cell somata as does the olfactory bulb. This is in contrast to the ventrally located subpallium where the entire ventral nucleus (Vv) and the ventral part of the dorsal nucleus (Vdv; separated by a red stippled line from Vdd in Figure 1B) of the ventral telencephalic area exhibit very many cell bodies stained for *islet1*-GFP with many stained fibers extending into the lateral neuropil and into the anterior commissure (ac; Figure 1A–D, 3B). Some of these subpallial fibers extend into the pallium, in particular a prominent projection arising in the ventral nucleus of the ventral telencephalon (Vv) that reaches the medial zone of the dorsal telencephalon (Dm; Figure 4C, 5A). Also, many of those fibers extend into the supracommissural nucleus (Vs) of the ventral telencephalon. More posteriorly, some *islet1*-GFP positive cell bodies are present in the ventral domain of the supracommissural nucleus of the ventral telencephalic area (Vs; Figure 3B). In contrast, there are no *islet1*-GFP positive cells in the lateral (Vl), central (Vc) (Figure 3A), postcommissural (Vp; Figure 1E, 3C) and intermediate nuclei (Vi) (Figure 3C) of the ventral telencephalon – the latter likely representing the homolog of the medial amygdala (see Biechl et al., 2017). However, the Vi receives a distinct *islet1*-GFP positive terminal field, apparently originating in the parvocellular preoptic nucleus (see below) (Figure 3C).

**FIGURE 5:**
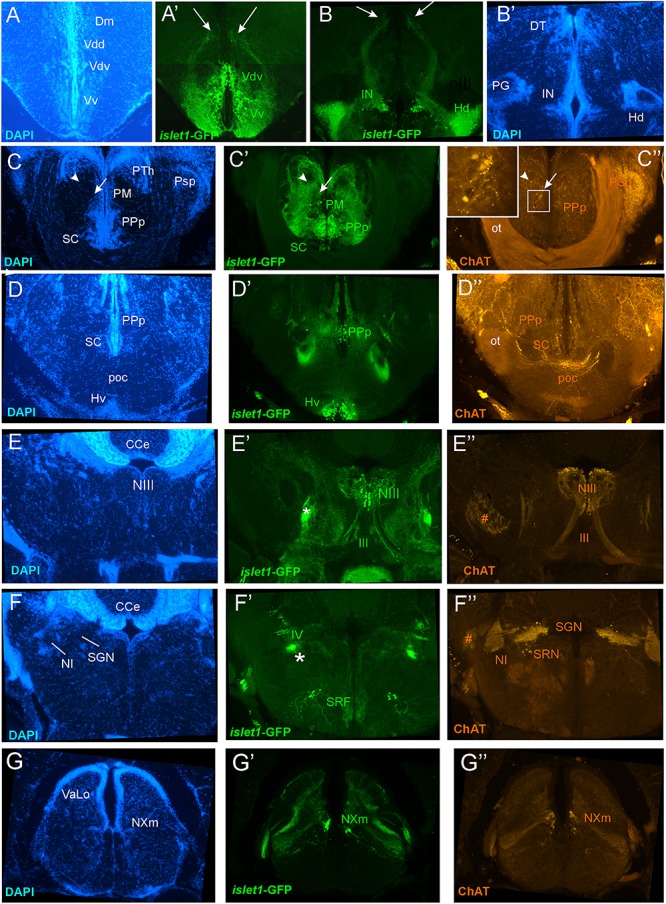
Details of *islet1*-GFP expression using fluorescent DAPI stain and choline acetyltransferase (ChAT) immunohistochemistry at 6 months of age. Conventions with lettering as in Figure 2. **(A,A’)** shows *islet1*-GFP positive axonal tract from the ventral nucleus of the ventral telencephalon (Vv) to the medial zone of the dorsal telencephalon (Dm). **(B,B’)** *islet1*-GFP axonal tract that projects from the intermediate hypothalamic nucleus (IN) to the dorsal thalamus. **(C,D)** Analysis of preoptic region at (**C**: DAPI, **C’**: *islet1*-GFP, **C”**: ChAT) level of magnocellular preoptic nucleus (PM) and (**D**: DAPI, **D’**: *islet1*-GFP, **D”**: ChAT) postoptic commissure (poc). Note that preoptic cholinergic cells are never double-labeled for *islet1*-GFP and strong cholinergic terminal field in the parvocellular superficial pretectal nucleus (PSp) originating in nucleus isthmi. Arrowhead in **(C)** indicates artifact (no DAPI cell nucleus seen). **(E–E”)** Magnification of midbrain tegmentum shows oculomotor nerve nucleus (NIII) and nerve exiting the brain (III). Asterisk points out hypothalamo-secondary gustatory nuclear tract. # indicates a ChAT positive fiber net (see Mueller et al., 2004). **(F–F”)** Region of secondary gustatory nucleus (SGN) shows ChAT cells in SGN and nucleus isthmi (NI) and superior reticular nucleus (SRN). Asterisk points out hypothalamo-secondary gustatory nuclear tract. # indicates a ChAT positive fiber net (see Mueller et al., 2004). **(G–G”)** Region of vagal sensory lobe (VaLo) and vagal motor nerve nucleus (NXm) shows some cholinergic vagal motor cells double-labeled for *islet1*-GFP. CCe, corpus cerebelli; Dm, medial zone of dorsal telencephalon; Hd/Hv, dorsal/ventral zone of periventricular hypothalamus; IN, intermediate hypothalamic nucleus; NI, nucleus isthmi; NIII, oculomotor nerve nucleus; NXm, vagal motor nerve nucleus; ot, optic tract; PM, magnocellular preoptic nucleus; poc, postoptic commissure; PPp, posterior parvocellular preoptic nucleus; PSp, parvocellular superficial pretectal nucleus; PTh, prethalamus; SC, suprachiasmatic nucleus; SGN, secondary gustatory nucleus; SRF, superior reticular formation; SRN, superior reticular nucleus; VaLo, vagal (sensory) lobe; Vdd/Vdv, dorsal/ventral subnucleus of dorsal nucleus of ventral telencephalon; Vv, ventral nucleus of the ventral telencephalon; III, oculomotor nerve; IV, trochlear nerve.

Beyond the subpallium, abundant *islet1*-GFP cell bodies are observed in all divisions of the preoptic region, that is, many in the anterior (PPa) and posterior parvocellular (PPp) preoptic, as well as some laterally located ones in the magnocellular (PM) preoptic and in the suprachiasmatic nuclei (SC; Figure 1D–G, 2A, 3C,D, 5C,D). As in the subpallium, the neuropil lateral to the preoptic region is densely stained. An interesting detail at preoptic levels relates to the ventral entopeduncular nucleus – the hypothesized homolog of the bed nucleus of the stria medullaris (ENv; Figure 1D,E; Mueller and Guo, 2009; but see also Turner et al., 2016). It clearly remains free of *islet1*-GFP cell body and fiber stain which is consistent with its being derivate from the embryonic eminentia thalami and not from the subpallium (Wullimann and Mueller, 2004; Mueller and Wullimann, 2009).

The posterior preoptic nucleus extends to the level of the ventral thalamus (prethalamus) which itself is *islet1*-GFP positive (VM/PTh; Figure 1F, 3D, 5C,D). More caudally, some *islet1*-GFP cells are seen in the anterior (A; Figure 2A), and also in the dorsal posterior (DP) and central posterior (CP) nuclei of the (dorsal) thalamus (Figure 1G–I). Notably, the periventricular pretectum (PPr) and all other pretectal nuclei remain free of *islet1*-GFP expression (Figure 1G–I, 2B). The dorsal accessory optic nucleus (DAO; Figure 1G), however, contains clearly *islet1*-positive somata and it appears that these cells migrate out from the periventricular nucleus of the posterior tuberculum (TPp; Figure 3E).

Regarding the basal plate of the diencephalon, sizable populations of *islet1*-GFP positive cells are present in the periventricular nucleus of the posterior tuberculum (TPp), but not in the paraventricular organ (PVO; Figure 1G–I, 3E). There are also no *islet1*-GFP cell bodies in the posterior tuberal nucleus (PTN), but strongly stained fiber masses surround this nucleus dorsally (Figure 1J, 2B–E, 3F,G). The preglomerular complex (PG) remains completely free of *islet1*-GFP cell bodies and fibers (Figure 1H,I, 2C,D). There are furthermore some *islet1*-GFP positive cells in the nucleus of the medial longitudinal fascicle (Nmlf; Figure 4H,I). *Islet1* immunopositive cells attributed to the sensory mesencephalic trigeminal nucleus have been reported in the 24 h embryonic zebrafish (Dyer et al., 2014), and possibly we see some *islet1*-GFP cells in the adult zebrafish brain (Figure 4I’).

In the hypothalamus, very many *islet1*-GFP cell bodies are present in the ventral periventricular hypothalamic zone (Hv; Figure 1G–I, 2B,C) and few such cells also extend into the lateral hypothalamic nucleus (LH; Figure 1I, 2B). Similarly, the dorsal periventricular hypothalamic zone (Hd) contains abundant *islet1*-GFP expressing cell bodies throughout the extent of the lateral recess (Figure 1H–J, 2B–I). Furthermore, the intermediate hypothalamic nucleus (IN; Figure 2B), wedged between posterior tuberal nucleus (PTN) and dorsal periventricular hypothalamic zone (Hd), contains *islet1*-GFP cells which appear to project to the dorsal thalamus (Figure 5B). Stained *islet1*-GFP fibers extend both from the ventral and dorsal hypothalamic periventricular zones into the laterally lying hypothalamic neuropil which overall gives the appearance of *islet1*-GFP expressing fibers outlining this portion of the hypothalamus. Notably, these stained hypothalamic fibers neither reach the nearby preglomerular complex (see above) nor the diffuse nucleus of the hypothalamic inferior lobe (DIL; Figure 1J,K, 2C–I) or the lateral torus (TLa; Figure 1H–J, 2B–E). Moreover, there are no *islet1*-GFP cell bodies in these three structures (PG, DIL, and TLa). The hypothalamic anterior tuberal nucleus (ATN) remains free of *islet1*-GFP fibers and cell bodies in most of its extent (Figure 1H, 2B), but some scattered cell bodies are present in its caudal part (Figure 1I). The pituitary (Pit) is another site with cells expressing *islet1*-GFP (Figure 1J, 2D,E). Finally, the unpaired anterior part of the hypothalamic caudal periventricular zone (Hc) shows many *islet1*-GFP cell bodies (Figure 2D, 3F,G), with dense fibers extending into the surrounding ventrolateral neuropil. In contrast, the posterior part of the caudal periventricular hypothalamus (Hc) which surrounds the posterior recess (PR) is completely free of *islet1*-GFP cell bodies, as is the corpus mamillare (Hc; CM; Figure 1J,K, 2E,F).

Reaching now the midbrain, the only *islet1*-GFP cell bodies are seen in the motor neurons of the oculomotor cranial nerve (NIII; Figure 1J, 2D,E, 5E) and – upon close inspection – in the Edinger–Westphal nucleus (NEW; Figure 4E) as well as in cells of a rostral perilemniscal nucleus (PLr; Figure 4E,G). In contrast, the alar plate midbrain, including the optic tectum (TeO) and the torus semicircularis (TS; Figure 1G–K, 2A–I), remains completely free of any *islet1*-GFP signal.

Turning finally to the rhombencephalon, distinct *islet1*-GFP positive terminals are present at the lateral edge of the interpeduncular nucleus (NIn; Figure 2F–H) and within the superior raphe (Figure 1K, 2I). Some *islet1-*GFP positive cell bodies are present in all hindbrain cranial nerve motor nuclei, i.e., the trochlear nucleus (NIVm; Figure 2F,G), two divisions each of the trigeminal (NVm; Figure 2J) and abducens (NVIm; Figure 2K,L) motor nuclei, as well as the facial (NVIIm; Figure 2L–O), glossopharyngeal (NIXm; Figure 2O) and vagal (NXm; Figure 1L, 2P, 5G) motor nuclei. Also the axons of these cranial nerve motor neurons (the motor roots) are positive for *islet1*- GFP. Furthermore, *islet1*-GFP is present in the two populations of octavolateralis efferent neurons in the very midline of the rhombencephalon (OENr/c; Figure 2L,N). Some scattered *islet1*-GFP positive cells are also seen in the superior reticular formation (SRF; Figure 1K, 2I, 5F) and in some cholinergic cells extending ventrally to the caudal perilemniscal nucleus (PLc, which itself is free of *islet1*-GFP) lying laterally to the lateral longitudinal fascicle (Figure 4F).

An *islet1*-GFP positive tract is most anteriorly seen to emerge from the *islet1*-GFP positive fiber mass lateral to the dorsal periventricular zone of the hypothalamus (Hd) and then runs laterally, bypassing the preglomerular complex (encircled with a red stippled line in Figure 2C), in caudal direction (asterisks in Figure 1–4) through the tegmental mesencephalon (Figure 2D,E) and into the rhombencephalon (Figure 2F–I). Finally, this tract is most caudally visible at the level where the trochlear root has decussated to the contralateral brain side and the (motor) trigeminal nerve is at its exit from the brainstem (Figure 1K, 2I). This is also the level of locus coeruleus and superior raphe (see below). Upon closer inspection, these *islet1*-GFP positive axons are seen to form a terminal field in the medial part of the secondary gustatory nucleus (Figure 4D). Thus, since these fibers likely originate in the dorsal periventricular hypothalamic zone (Hd), they apparently form a hypothalamo-secondary gustatory nuclear tract (see section “Discussion”).

Finally, we observe *islet1*-GFP positive cell bodies in the spinoccipital region which is transitory between the most caudal ventral hindbrain and the spinal cord (Figure 1M). Some scattered *islet1*-GFP cells are also present at the edge of the catecholaminergic area postrema (AP; Figure 1M). These cells likely represent most dorsally located vagal motor neurons. Even more caudally, in the area of the viscerosensory commissural nucleus of Cajal (NC), a strong *islet1*-GFP positive fiber crossing is seen in this nucleus (commissura infima of Haller; Figure 2Q), but no *islet1*-GFP cell bodies. However, *islet1-*GFP cell bodies are present in the ventral horn of the spinal cord (VH) itself where they give rise to ventral (motor) roots (vr; Figure 2Q).

### Double-Label of Tyrosine Hydroxylase and *islet1*-GFP

Immunohistochemical visualization of tyrosine hydroxylase (TH) is used here for two reasons: (1) TH provides for well investigated landmarks in the zebrafish brain, in particular in the forebrain (Ma, 1994a,b, 1997; Kaslin and Panula, 2001; Rink and Wullimann, 2001; Yamamoto et al., 2011) and, thus, supports the present neuroanatomical analysis. (2) Detailed analysis of TH cell groups co-expressing *islet1*-GFP informs us about their likely early developmental dependence on upstream *sonic hedgehog* (*shh*) signaling.

For this analysis, the adult zebrafish brain distribution of TH positive cells (Figure 1A3–M3, right column) is compared to DAPI and *islet1*-GFP stains of the same sections already described in the previous section. Table 2 provides an overview on all CNS structures labeled for TH, *islet1*-GFP or both. As expected, olfactory bulb TH positive cells remain single-labeled because no *islet1*-GFP cells are present there (Figure 1A). The rostrocaudally extensive TH cell population on the lateral edge of the series of subpallial nuclei (Vv, Vd, Vs; Vp) in the ventral telencephalon (SD; Figure 1B3–E3, 3A’) lies notoriously close to or even intermingles with the masses of *islet1*-GFP cells seen in these subpallial nuclei. However, there is no overlap of the two markers because subpallial TH cells never express *islet1*-GFP (Figure 1B3–E3, 3A’).

In contrast, the preoptic region exhibits many cells in which the two markers overlap, for example in the anterior and posterior periventricular preoptic nuclei (PPa, PPp; Figure 1D3–F3, 3C,D). The PPp exhibits TH cells in its anterior part (Figure 1F2, 3D) but lacks such cells in its most posterior extent (Figure 1G2). In contrast, both the magnocellular preoptic (PM) and the suprachiasmatic nuclei (SC) show no double-labeled cells (Figure 1F3, 3D). Double-label of TH and *islet1*-GFP also exists in ventromedial tier cells of the ventral thalamus (prethalamus) (VM; Figure 1F3). These dopaminergic cells correspond to the mammalian zona incerta (Wullimann and Rink, 2001). The dopaminergic periventricular pretectal cells remain completely free of *islet1*-GFP (PPr; Figure 1G3–I3).

In the posterior tuberculum, the large (magnocellular) pear-shaped TH cells of the posterior tuberculum (TPp-m; Figure 1G3–I3) and the TH cells of the paraventricular organ (PVO; Figure 1H3,I3) are never double-labeled with *islet1*-GFP. However, some small (parvocellular) TH cells of the periventricular nucleus of the posterior tuberculum (TPp-p; Figure 1I3; note left inset from another specimen, and Figure 3E,E’) are sometimes double-labeled. In contrast, the posterior tuberal nucleus TH cells (PTN; Figure 1J3, 3F) are always negative for *islet1*-GFP, but densely surrounded dorsally by *islet1*-GFP positive fibers.

In the hypothalamus, the *islet1*-GFP positive intermediate nucleus (IN; Figure 2B) is known to contain dopaminergic cells, but their synthesis pathway uses TH2 (not TH1) which is only weakly, if at all, visualized by TH immunohistochemistry (Yamamoto et al., 2010, 2011). Thus, no decision whether these TH cells are double-labeled with *islet1*-GFP can be made. The posterior part of the caudal periventricular hypothalamus around the posterior recess (Hc) is free of *islet1*-GFP as already mentioned. Thus, its TH stained cells (which also express mostly *TH2* and are therefore weakly immunostained; see above comment on IN) cannot be double-labeled (Hc; Figure 1J3). However, the anterior part of the caudal hypothalamic periventricular zone (Hc; Figure 2D, 3F,G) contains numerous *islet1*-GFP cells. In our preparations, we see no immunostained TH cells in this anterior part of Hc (but see Rink and Wullimann, 2001). However, because the latter expresses *TH2* (Yamamoto et al., 2011; note their Figure 12D), dopamine cells are present there, but again (as in IN) cannot be checked for double-label. The strongly immunostained TH cells dorsal to the anterior Hc are never double-labeled and are known to express *TH1* and not *TH2* (Yamamoto et al., 2011) and we interpret them as the most caudal tip of the posterior tuberal nucleus (PTN; Figure 1J3, 3F,G).

In the rhombencephalon, the (TH positive) locus coeruleus (Figure 1K3) and the area postrema (Figure 1M3) are free of *islet1*-GFP cell bodies. However, there are a few *islet1*-GFP/TH double-labeled cells at the dorsal border of the vagal motor nucleus (Figure 1L3; the vagal group of catecholaminergic neurons; Ma, 1997).

### Double-Label of Choline Acetyltransferase and *islet1*-GFP

For this analysis, the adult zebrafish brain distribution of choline acetyltransferase cells (ChAT; Figure 2A3–Q3) is compared to DAPI (Figure 2A1–Q1) and *islet1*-GFP (Figure 2A2–Q2) stains of the same sections already described above. Table 3 provides an overview on all CNS structures labeled for ChAT, *islet1*-GFP or both. The most anterior ChAT positive neurons are in the preoptic region (PPa, PM, PPp, and SC) and are always *islet1-*GFP negative (Figure 2A3, 4A,B, 5C,D). Two minor ChAT cell populations, one in the dorsal thalamus and one close to the preglomerular complex (Figure 2B3, 4H), also both remain *islet1*-GFP negative. The cholinergic rostral tegmental nucleus (RT of Grover and Sharma, 1981; Figure 2C3), which forms one of several cholinergic inputs to the optic tectum (Mueller et al., 2004), is also free of *islet1*-GFP. The most prominent *islet1*-GFP positive cell bodies in the tegmental midbrain are those of the (cholinergic) oculomotor cranial nerve (NIII; Figure 2D3,E3). Additionally, we found overlap of ChAT and *islet1*-GFP in the Edinger–Westphal nucleus (Figure 4E,E’). In sharp contrast, the abundant cholinergic cells seen in the periventricular gray zone of the optic tectum are *islet1*-GFP free over their entire anteroposterior extent (PGZ; Figure 2A3–I3). In fact, the entire midbrain roof, including torus longitudinalis, optic tectum and torus semicircularis is completely free both of *islet1*-GFP positive and of cholinergic cells (except for the cholinergic cells in the PGZ just mentioned).

Continuing with the hindbrain, the motor nuclei of all cranial nerves, that is, the trochlear (NIV; Figure 2F3,G3) and two divisions each of trigeminal (NV; Figure 2J3) and abducens (NVI; Figure 2K3,L3), as well as the facial (NVII; Figure 2L3–O3), glossopharyngeal (NIX; Figure 2O3) and vagal nerve motor nuclei (NX; Figure 2P3, 5G) light up immunohistochemically for ChAT. Additionally, ChAT positive cells are seen in octavolateralis efferent neurons which form two compact groups in the general area of the facial motor nerve nucleus in the midline immediately dorsal to the medial longitudinal fascicle (OENr/c; Figure 2L3,N3). Many, but not all, of these motor cholinergic neurons express *islet1*-GFP in each of those structures. Finally, viscero- and somatomotor neurons in the ventral horn of the spinal cord are ChAT positive (Figure 2Q3) and are partly also *islet1*-GFP positive.

Thus, overall, *islet1*-GFP expression in the zebrafish hindbrain is strictly limited to cholinergic cranial nerve motor nuclei and efferent cells of the octavolateralis system. A notable exception are the cholinergic cells ventral to the caudal perilemniscal nucleus (PLc; Figure 4F) which are also labeled for *islet1*-GFP. The PLc itself is also cholinergic, but shows no *islet1*-GFP. In the spinal cord, a fraction of ventral horn motor neurons and their ventral root fibers is double-labeled for *islet1*-GFP and ChAT (Figure 2Q).

In contrast, the remaining hindbrain cholinergic systems, i.e., nucleus isthmi, nucleus lateralis valvulae and secondary gustatory nucleus as well as the superior reticular nucleus (Figure 2G3–I3, 5F) are completely free of *islet1*-GFP (for delimitation of these cholinergic structures see Mueller et al., 2004; Castro et al., 2006b; Yáñez et al., 2016). Furthermore, all hindbrain primary sensory nuclei and all parts of the cerebellum remain free of *islet1*-GFP.

We also investigated a 6 month-old zebrafish brain for *islet1*-GFP and ChAT. In general, the *islet1*-GFP expression pattern turned out to be qualitatively identical to the 3 months brain; some *islet1*-GFP structures and interesting co-localization issues reported above for the 3 months zebrafish brain are shown at 6 months in Figure 5 and will be discussed below.

### Conspicuous *islet1*-GFP Positive Terminal Fields

There are some very obvious axonal projection patterns visible in *islet1*-GFP sections of the adult zebrafish brain which we shortly summarize here for better visibility.

#### Telencephalon

A distinct projection arises from the ventral nucleus of the ventral telencephalon (Vv) and terminates in the medial zone of the dorsal telencephalon (Dm; Figure 4C, 5A) which comparatively would correspond to a septo-amygdalar projection. A distinct *islet1*-GFP positive tract apparently originating in the anterior preoptic region (PPa) is seen to form a terminal field in the intermediate nucleus of the ventral telencephalon (Vi; Figure 3C), which likely represents the medial amygdala homolog (Biechl et al., 2017).

#### Diencephalon

Another distinct projection of *islet1-*GFP positive axons is seen to run from the intermediate hypothalamic nucleus (IN) to the dorsal thalamus (Figure 4I, 5B).

#### Rhombencephalon

The prominent hypothalamo-secondary gustatory nucleus tract (Figure 2C2–I2, 4D) runs from the dorsal periventricular hypothalamus to the lateral part of the secondary gustatory nucleus (see above). The fibers at the lateral edges of the interpeduncular nucleus (NIn) and in the superior raphe (SR) might originate in the ventral telencephalon because a similar efferent projection pattern to these nuclei has been described following ventral telencephalic tracer injections (Rink and Wullimann, 2004). The fibers in the nucleus commissuralis of Cajal might originate in vagal motor neurons, and/or neurons in the dorsal periventricular hypothalamic zone and/or in the preoptic region because all three have been shown to project to NC (Uezono et al., 2015) and to contain many *islet1*-GFP positive cells (see above).

### Larval *sonic hedgehog* Expression

**FIGURE 6:**
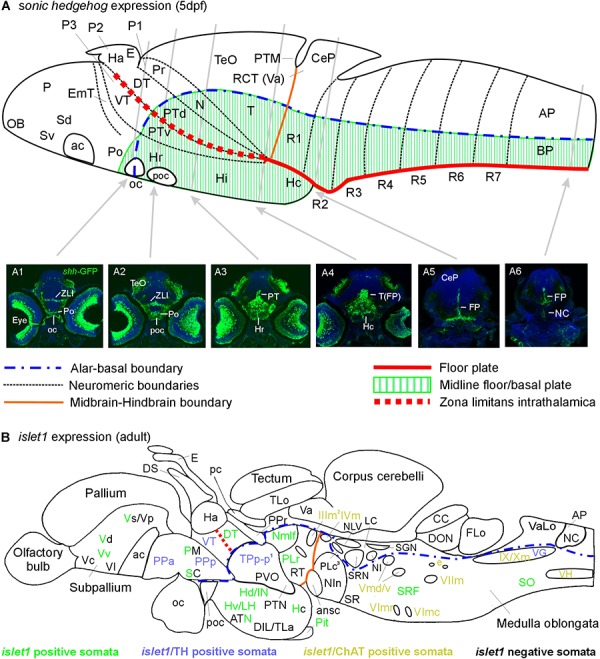
Schematic sagittal sections of a larval **(A)** and an adult **(B)** zebrafish brain. **(A)** summarizes *sonic hedgehog*-GFP expression at the larval stage (4–5 days) in floor plate of midbrain/hindbrain and forebrain basal/alar plate as described previously (Biechl et al., 2016). **(A1–A6)** Transverse sections illustrate *shh*-GFP expression at levels indicated. Note that at larval stages, the most anterior *sonic hedgehog*-GFP expression site is in the alar plate preoptic region and that there is no additional basal telencephalic expression. **(B)** shows adult *islet1*-GFP expression (color letters) versus structures negative for this gene expression (black letters) as established in the present study. See figure for color code of *islet1*-GFP structures either singly labeled or double-labeled for tyrosine hydroxylase or choline acetyltransferase in addition. Structures with *islet1*-GFP cells in a restricted subarea are shown using black and colored letters. Overall, *islet1*-GFP expressing structures are in the basal plate, except for the preoptic region, the thalamus and the subpallial telencephalon (see text for more information). (1) Note that the large posterior tubercular pear-shaped dopaminergic neurons (TPp-m) projecting to basal ganglia (Vd) are *islet1*-GFP negative. Note furthermore that *islet1*-GFP cells of the dorsal accessory optic nucleus appear to originate in TPp-p. (2) Note that also the visceromotor Edinger–Westphal nucleus is double-labeled. (3) Note that cholinergic cells ventrolateral to the also cholinergic caudal perilemniscal nucleus (PLc) express *islet1*-GFP. ac, anterior commissure; ansc, ansular commissure; AP, alar plate in **(A)** and area postrema in **(B)**; ATN, anterior tuberal nucleus; BP, basal plate; CC, crista cerebellaris; CCe, corpus cerebelli; CeP, cerebellar plate; DIL, diffuse nucleus of inferior lobe; DON, descending octaval nucleus; DS, saccus dorsalis; DT, dorsal thalamus; e, octavolateralis efferent neurons; EmT, eminentia thalami; FLo, facial (sensory) lobe; FP, floor plate; Ha, habenula; Hc/Hd/Hv, caudal/dorsal/ventral zone of periventricular hypothalamus; Hi/Hr (larval), intermediate/rostral hypothalamus; IN, intermediate hypothalamic nucleus; LC, locus coeruleus; LH, lateral hypothalamic nucleus; N, area of nucleus of medial longitudinal fascicle; NC, notochord in A6, commissural nucleus of Cajal in **(B)**; NI, nucleus isthmi; NIn, nucleus interpeduncularis; NLV, nucleus lateralis valvulae; OB, olfactory bulb; oc, optic chiasma; P, pallium; pc, posterior commissure; Pit, pituitary; PLc/PLr, caudal/rostral perilemniscal nucleus; PM, magnocellular preoptic nucleus; Po, preoptic region; poc, postoptic commissure; PPa/PPp, anterior/posterior parvocellular preoptic nucleus; Pr, pretectum; PTd/PTv, dorsal/ventral posterior tuberculum; PTM, posterior tectal membrane; PTN, posterior tuberal nucleus; PVO, paraventricular organ; R1–R7, rhombomeres 1–7; RCT, rostral cerebellar thickening; SC, suprachiasmatic nucleus; SCm, spinal cord motor cells; Sd/Sv (larval), dorsal/ventral subpallium; SGN, secondary gustatory nucleus; SO, spino-occipital region; SR, superior raphe; SRF, superior reticular formation; SRN, superior reticular nucleus; T, midbrain tegmentum; TeO, optic tectum; TLa, torus lateralis; TLo, torus longitudinalis; TPp-p, parvocellular cell part of periventricular posterior tubercular nucleus; Va, valvula cerebelli; VaLo, vagal (sensory) lobe; Vc/Vd/Vp/Vs/Vv, central/dorsal/posterior/supracommissural/ventral nucleus of ventral telencephalon; VG, vagal group of catecholamine neurons; VH, spinal ventral horn motor cells; VT, ventral thalamus; ZLI, zona limitans intrathalamica; IIIm, oculomotor nerve nucleus; IVm, trochlear motor nerve nucleus; IXm, glossopharyngeal motor nerve nucleus; Vmd, dorsal trigeminal motor nerve nucleus; Vmv, ventral trigeminal motor nerve nucleus; VImc/VImr, caudal/rostral abducens motor nerve nucleus; VIImc/NVIImr, caudal/rostral facial motor nerve nucleus; IX/NXm, glossopharyngeal/vagal motor nerve nucleus.

Because the signaling molecule Sonic hedgehog (SHH) is involved in ventralizing the developing neural tube (see section “Introduction”), we provide here a short description of this gene’s larval expression using a well established *shh-*GFP line (Shkumatava et al., 2004; Biechl et al., 2016). In a previous report, we documented *shh*-GFP expression at larval stages in detail and focused in particular on cerebellar *shh*-GFP cells (Biechl et al., 2016). Here we give selected transverse sections of a *shh*-GFP specimen along the entire neuraxis of the 5 dpf zebrafish brain (Figure 6A). This allows to recognize the SHH signaling centers in the larval brain known to initiate *islet1* expression in developing neurons nearby.

Beginning posteriorly, the prominent few cells of the floor plate (FP) are labeled and seen to extend radial fibers toward the ventral pial periphery where they form a broad endfeet mesh in the spinal cord (Figure 6A6) and hindbrain (Figure 6A5). In the mesencephalic tegmentum (T), these floor plate cells are more broadly distributed also laterally and ventrally in the midline (Figure 6A4). The caudal hypothalamus is deflected caudally becoming located below the midbrain and numerous *shh*-GFP cells are seen in it. More anteriorly, *shh*-GFP cells are present in the basal diencephalon, i.e., the posterior tuberculum (PT; Figure 6A3), with more such cells in the rostral hypothalamus (Hr) anterior to the PT. Finally, *shh-*GFP positive cells are seen in the zona limitans intrathalamica (ZLI; Figure 6A2) and in the preoptic area (Po; Figure 6A1,A2). In the eyes, various cell types express *shh*-GFP, including ganglion cells; this is the reason for fiber reactivity seen in the optic nerve and in visual layers within the optic tectum which is otherwise mostly free of *shh*-GFP cell bodies (but see Biechl et al., 2016).

## Discussion

### *islet1* and *islet1*-GFP During Zebrafish Brain Development

The pioneering work of Hitoshi Okamoto and colleagues revealed three LIM/homeodomain *islet* genes in the zebrafish, *islet1*, *islet2*, and *islet3* (Inoue et al., 1994; Tokumoto et al., 1995; Kikuchi et al., 1997; Hirate et al., 2001; Segawa et al., 2001; Okamoto et al., 2004; Uemura et al., 2005). These and additional studies showed that *islet1* and *islet2* are expressed in the embryo (15–36 h) in non-overlapping sets of segmentally repeated rostral versus caudal subpopulations of motor neurons, respectively, in embryonic zebrafish spinal cord, but that *islet3* is not expressed in motor neurons (Korzh et al., 1993; Appel et al., 1995; Tokumoto et al., 1995; Thor et al., 1999; Segawa et al., 2001; Hutchinson and Eisen, 2006; Seredick et al., 2012). Analyses of the *islet* gene family revealed that zebrafish have only these three *islet* genes (Gong et al., 1995; Tokumoto et al., 1995), but that salmonids (such as rainbow trout and Chinook salmon) have two paralogs of *islet1* and *islet2* each, i.e., *islet1a* & *b* and *islet2a* & *b* (Gong et al., 1995). However, more recently, zebrafish *islet2* and *islet3* were redefined as two paralogs of *islet2* (*a* and *b*) of which only *islet2a* is specifically expressed in subsets of caudal motor neurons (Pittman et al., 2008; Aoki et al., 2014; Moreno R.L. et al., 2018) while *islet2b* is expressed in zebrafish retinal ganglion cells (Pittman et al., 2008).

Some studies also revealed additional expression of *islet1* in forebrain domains and segmental hindbrain clusters (rhombomeres 1–7), but this was not investigated in enough detail (Inoue et al., 1994; Tokumoto et al., 1995). Furthermore, *islet 2* and *islet3* (i.e., *islet2a* and *islet2b*, see above) genes are additionally expressed in retina (*islet3*) and optic tectum (both) and in the trigeminal ganglion (Inoue et al., 1994; Tokumoto et al., 1995).

Later, an *islet1*-GFP line was created by fusing GFP sequences to *islet1* promotor/enhancer sequences, Tg(islet1:gfp)rw0, sufficient for neural specific expression in cranial motor neurons (Higashijima et al., 2000). The latter develop between 28 h and 4 days to their full extent (motor nuclei of cranial nerves III, IV, V, VII, IX, X), with those of VII, IX, X, and octavolateralis efferent neurons (OEN) appearing latest. Although the two abducens motor nuclei (VI) were not mentioned in this study, they were likely overlooked because of their smallness. A comparison of *islet1*-GFP to *islet1*-mRNA expression revealed early (28–40 h) more *islet1* than *islet1*-GFP expressing cells in motor nuclear regions of nerve III, IV, and VII, speaking for an ongoing refinement there. One singular qualitative difference in expression is that the trigeminal sensory ganglion is seen in *islet1 in situ* hybridization, but not in *islet1*-GFP assays. Furthermore, Dyer et al. (2014) have reported Islet1 immunopositive cells in the epiphysis which do not show up in the *islet1*-GFP line. However, *islet1*-GFP is present in all other branchiomeric cranial nerve ganglia (VII, IX, and X). From 4 days on, i*slet1*-GFP cells other than motor neurons appear in addition in the forebrain, clearly identifiable when checking with our data as subpallium, preoptic region and dorsal periventricular hypothalamic zone (see section “Results”). Thus, the *islet1*-GFP line has been validated by their creators to faithfully show *islet1* expression and not to express GFP in additional domains.

In our study on adult zebrafish brains, we see qualitatively a highly similar picture. However, because of our neuroanatomical focus and methodology, we identify structures in much more detail (see section “Results” summarized in Figure 6B). Furthermore, we noted that in 3 as well as in 6 months old zebrafish *islet1*-GFP cells are absent in any branchiomeric sensory cranial nerve ganglia which speaks for a downregulation of *islet1*-GFP in the adult zebrafish peripheral nervous system.

### *islet* Gene Expression in Zebrafish Compared to Other Vertebrates

Tetrapod vertebrates have *islet1* and *islet2* genes expressed in different sets of spinal and rhombencephalic motor neurons (Tsuchida et al., 1994; Ericson et al., 1995; Pfaff et al., 1996; Varela-Echavarría et al., 1996; Guidato et al., 2003; Ju et al., 2004; Showalter et al., 2004; Thaler et al., 2004), but they lack an *islet3* (*islet2b*; see above) gene generally (Gong et al., 1995; Tokumoto et al., 1995). Thus, it would appear that *islet* genes were duplicated during the 3rd Whole Genome Duplication (3rd WGD) at the base of teleost phylogeny (Amores et al., 1998; Postlethwait et al., 2004). Among teleosts, salmonids (rainbow trout, Chinook salmon) additionally express even two paralogs of both *islet1* and *islet2* each (see above) which is attributed to tetraploidy special for salmonids and not the 3rd WGD (Gong et al., 1995).

Since lampreys seem at least to have one *islet* gene expressed in motor neurons (Osório et al., 2005; Kim et al., 2015), possibly the divergence into *islet1* and *islet2* originated with the 2nd WGD (Ohno, 1970; Sidow, 1996; Panopoulou and Poustka, 2005) at the base of the vertebrate tree.

While studies on tetrapods never report *islet2* expression in the forebrain, they do so for *islet1*. For example, already Ericson and colleagues (1995) in their seminal paper state that chick *islet-2* mRNA is neither expressed in rhombencephalon and mesencephalon, nor in diencephalon or telencephalon (but see below for later detection of *islet2* in rhombencephalon).

We focus the following comparative discussion on the telencephalon. The hypothalamus also strongly expresses *islet1* in zebrafish as in tetrapods but this issue has been addressed in comparative terms in a recent paper from our lab (Herget et al., 2014). In anamniote tetrapods such as amphibians, like frogs (Moreno et al., 2008a,b,c; Domínguez et al., 2010 and newts: Moreno N. et al., 2018) or sarcopterygian lungfish (González et al., 2014; Moreno N. et al., 2018), *islet1* is expressed in most of subpallium, in particular in septum, striatum (strongly), pallidum (more weakly), and central amygdala, but not in the medial amygdala and not in the most dorsal part of the striatum.

Regarding amniotes, telencephalic *islet1* expression in rodents is restricted at embryonic day 15.5 in the mouse to basal ganglia and septum, particularly strongly the lateral ganglionic eminence (LGE) (Long et al., 2009), except for a most dorsal LGE *islet1* free subpopulation migrating into the olfactory bulb (Stenman et al., 2003). Thus, *islet1* is indispensable for intrinsic striatal neuronal development in amniotes (Stenman et al., 2003; Flames et al., 2007; Long et al., 2009; Medina et al., 2014). However, the rodent pallidum has *islet1* expressing cells (seen in the medial ganglionic eminence, MGE) likely due to ventrally migrating LGE cells between subventricular zone and mantle zone (Nóbrega-Pereira et al., 2010; Bupesh et al., 2011, 2014; Medina et al., 2014) and these pallidal *islet1* cells in the adult brain project back to striatum (Medina et al., 2014). A similar situation considering the strong *islet1* expression in striatum and weak one in pallidum seems to exist in reptiles and birds as well as in amphibians (Moreno et al., 2010, 2012; Medina et al., 2014). In birds, *islet1* is similarly expressed in embryonic forerunners of septum, pallidum and striatum, again with a region of most dorsal striatum free for it (Abellán and Medina, 2009). In the amygdala, Bupesh et al. (2011), Kuenzel et al. (2011), and Medina et al. (2011) report *islet1* positive cells in central amygdala, possibly also invading it from LGE. These reports also show that *islet1* is expressed in central, but not in medial amygdala. These data in tetrapods conform well with our findings of *islet1* expression in septum (i.e., Vv), basal ganglia (Vd) and central amygdala (Vs) homologs in zebrafish.

A previous study reporting on adult *islet1* zebrafish forebrain expression (Ganz et al., 2012) is in general agreement with our findings, but we disagree with its interpretation of data. We see clearly *islet1* expression in the ventral division of Vd (see section “Results”) as well as in Vv whereas Ganz et al. (2012) interpret all of the subpallial *islet1* domain as septum (Vv) which seems odd in both comparative and developmental terms. The likely pallidostriatal teleost homolog Vd has been shown by *GAD67 in situ* hybridization stain in its entire morphological distinct outline bordered ventrally by (equally *GAD67* positive) septal homolog Vv and dorsally by (*GAD67* negative) pallial division Dm in the adult zebrafish brain (Mueller and Guo, 2009). Compared to these stains, our *islet1-*GFP stain clearly covers Vv, but also the ventral part of Vd (Vdv). This ventral division of Vd has been substantiated with differential gene expression in larval zebrafish before as corresponding to the pallidal part of the subpallium because of expression of *lhx6* and *lhx7-* both diagnostic for early pallidum, but not striatum, also in tetrapods (amniotes) (Mueller et al., 2008; Mueller and Wullimann, 2009; Wullimann, 2009; see discussion there). Thus, we agree with González et al. (2014) on the presence of a common bauplan of vertebrate basal ganglia and their description of separate pallidal and striatal parts in teleost Vd and in particular with their observation that *islet1* expression does not reach up to the pallial boundary. However, data on *lhx6/7* are not available for the adult zebrafish brain and we can thus not know whether these genes are still expressed similarly to the larval brain within the adult Vd. Additional rather extensive studies on GABAergic cell markers in our lab furthermore indicate that this pallidal teleost embryonic division is the origin of most if not all of the GABA cells invading the pallium as similarly observed in amniotes (Wullimann and Mueller, 2002; Mueller and Wullimann, 2003; Mueller et al., 2006; reviewed in Mueller and Wullimann, 2016).

The lack of *islet1* expression in the zebrafish subpallial division called Vdd contrasts with strong expression in the Vdv division (see section “Results”). This is puzzling, because, as discussed in the previous paragraph, Vdd is the homolog of the strongly *islet1* positive tetrapod striatum proper (or mammalian LGE developmentally spoken) whereas the Vdv corresponds to the tetrapod pallidum (or mammalian MGE developmentally spoken). Thus, another *islet* gene paralog may be expressed in the dorsal part of the dorsal nucleus and maybe in other *islet1*-GFP free subpallial zebrafish ventral telencephalic nuclear parts (such as the dorsal part of Vs) similar to the subfunctionalization between *islet1* and *islet* 2a described for the spinal cord and hindbrain motor neuron development in all vertebrates (see above). Alternatively, there might be a downregulation of *islet1* in adult zebrafish stages in these subpallial *islet1*-free areas (i.e., Vdd and dorsal part of Vs) which is particularly likely if *islet1* positive cells should originate in Vdd and migrate out.

### Ventralization Along the Zebrafish Neuraxis as Seen With *shh*-GFP, *islet1*-GFP, and ChAT

#### Hindbrain and Midbrain

It is well established in vertebrates that the floor plate emits the morphogen Sonic hedgeghog (SHH) along the entire neuraxis (including spinal cord and brain up to the anterior end of the midbrain; see Figure 6A) and hereby induces motor neurons in dorsolaterally adjacent basal plate regions (see section “Introduction”). One consequence of this induction is the expression of *islet1* in these future motor neurons (see section “Introduction”). In line with this textbook knowledge, we still observe *islet1*-GFP positive neurons in the ventral horn of the spinal cord and in all cranial nerve motor nuclei of the 3 and 6 months adult zebrafish brain of *islet1*-GFP line specimens (Figure 2, 6B). Notably, this does not apply to each and every motor neuron, as can be deduced from a double-label approach visualizing ChAT in brain sections of such transgenic fish (see section “Results”). However, as discussed above, the work of Hitoshi Okamoto and colleagues clarified the situation regarding the expression and complementary roles of *islet 1 and 2 g*enes in the zebrafish spinal cord. Highly likely a similar subfunctionalization of *islet* genes is active in vertebrate hindbrain motor nuclei. Indeed, chicken motor nuclei III, IV VI, and XII depend on *islet1* and *islet2* (Varela-Echavarría et al., 1996; Guidato et al., 2003).

Most remaining (i.e., non-motor) cholinergic structures in the zebrafish brain remain negative for *islet1*-GFP. These include in the hindbrain the nucleus lateralis valvulae (which possibly has a midbrain contribution), the secondary gustatory nucleus, the caudal perilemniscal nucleus, nucleus isthmi, the superior reticular nucleus and the midbrain rostral tegmental nucleus, but also all minor cholinergic forebrain populations are *islet1*-GFP free, such as cells in the preoptic region, dorsal thalamus and near the preglomerular complex (Figure 2, 4, 5, 6B). The few cholinergic and *islet1*-positive cells ventrolateral to the cholinergic, but *islet1*-GFP negative caudal perilemniscal nucleus (Figure 4F) represent a peculiar exception.

In line with their generally more dorsal locations and sensory or integrative related functions, these non-motor cholinergic systems are corroborated by lack of *islet1*- GFP expression as belonging to the alar plate. This conclusion is further supported by the fact that other dorsal structures – for which there can be no doubt about their being alar plate derivatives – remain completely free of cell body stain for *islet1*-GFP. Among these *islet1*-GFP free structures are all primary sensory nuclei, such as the commissural nucleus of Cajal, the medial funicular nucleus, the vagal, glossopharyngeal and facial lobes, the octaval and lateral line sensory nuclei, the sensory trigeminal column, as well as higher order multisensory (optic tectum, torus semicircularis, and torus longitudinalis) and sensorimotor integrative structures (e.g., all cerebellar divisions), all of which without exception remain free of *islet1*-GFP cell bodies (Figure 6B). This suggests that only basal plate (but not alar plate) cholinergic midbrain and hindbrain motor systems (plus some non-cholinergic cells in the superior reticular formation, rostral perilemniscal nucleus, spino-occipital region and the vagal catecholamine group; Figure 1, 2, 4E, 6B) are dependent on SHH signaling. The analysis further shows that apart from motor neurons (plus some cholinergic cells ventrolateral to the caudal perilemniscal nucleus and the non-cholinergic systems just mentioned), additional hindbrain/midbrain basal plate populations do not depend on *shh-islet1* activity.

#### Forebrain

Regarding the developing vertebrate forebrain (including the zebrafish), *sonic hedgehog* continues to be expressed basally, namely in the basal plate of the posterior diencephalon, in particular basal parts of prosomere 1 (i.e., the region of the nucleus of the medial longitudinal fascicle), through prosomeres 2 and 3 (the posterior tuberculum in the zebrafish) and in parts of the basal plate hypothalamus (Figure 6A). In addition, *sonic hedgehog* expression extends into ventrocaudal domains of the larval zebrafish forebrain alar plate, namely, the preoptic region (Figure 6A). Also, in all vertebrates, the zona limitans intrathalamica (ZLI), which forms the developmental transverse boundary between thalamus (P2) and ventral thalamus (prethalamus; P3) expresses *shh* (Figure 6A). How is this early *shh* expression reflected in *islet1*-GFP expression in the adult zebrafish forebrain?

#### Basal Plate Posterior Diencephalon

In addition to the midbrain/hindbrain basal plate elements just discussed, various basal plate derivatives of the posterior diencephalon (P1-3) stain for *islet1*-GFP. Strongly stained *islet1*-GFP populations are present in the periventricular nucleus of the posterior tuberculum (TPp). These cells may also be double-labeled by TH (Figure 6B). However, both the paraventricular organ (PVO) and posterior tuberal nucleus (PTN), as well as the more migrated posterior tubercular populations seen in the preglomerular nuclear complex remain free of *islet1*-GFP. Some *islet1*-GFP positive somata are furthermore present in the nucleus of the medial longitudinal fascicle. Thus, there are at least some *islet1*-GFP cells in all three basal plate divisions of the posterior diencephalon.

#### Alar Plate Posterior Diencephalon

Another site of interest in the posterior diencephalon is the zona limitans intrathalamica (ZLI). In all vertebrates examined, this transverse boundary between dorsal and ventral thalamus (i.e., thalamus and prethalamus) expresses *sonic hedgehog* during development (Figure 6A). This expression domain is aberrant in comparison to this gene’s basal longitudinal expression seen in the rest of the brain because the ZLI is a transverse structure. Thus, SHH is issued in a position here to act both in rostral and caudal direction within the alar plate rather than in the usual ventrodorsal direction along the neuraxis. Consequently, we observe adult *islet1*-GFP positive cells both in dorsal and ventral thalamic nuclei (thalamus/prethalamus; Figure 6B) which clearly belong to the alar plate. Like the dopaminergic cells of the periventricular nucleus of the posterior tuberculum (TPp), also those of the prethalamus (PTh or VT) are TH/*islet1*-GFP double-labeled. However, no *islet1-*GFP positivity at all is seen in the pretectum – and, thus, also not in its large population of dopamine cells – whose cells apparently are not influenced by SHH, presumably because of its remoteness to the ZLI. These facts nicely explain in detail this exceptional extraterritorial occurrence of *islet1* expression in zebrafish diencephalic alar plate derivatives (DT, VT) and further show that – when present – diencephalic TH/dopamine cells are included in this effect (TPp, VT).

#### Hypothalamus

The basal plate of the anterior forebrain (secondary prosencephalon) generally shows broad expression of *islet1*-GFP in the transgenic line. Main *islet1*-GFP expression domains are present in the ventral and dorsal zones of the periventricular hypothalamus (Hv, Hd) and in the lateral hypothalamic nucleus (LH). The intermediate hypothalamic nucleus (IN) contains many *islet1*-GFP cells whereas the anterior tuberal nucleus (ATN) contains only some *islet1*-GFP positive cells caudally, but not anteriorly. Finally, the pituitary expresses *islet1*-GFP. Furthermore, only the anterior part of the caudal periventricular zone of the hypothalamus (Hc) shows strong *islet1*-GFP positivity, but the posterior part of Hc surrounding the posterior recess is free of *islet1*-GFP expression (Figure 6B).

Overall this suggests that most of the periventricularly derived basal plate zebrafish hypothalamus is dependent on *islet1* expression as would be expected from the broad early *shh* expression in these regions (Figure 6A). For example, *islet1* has a crucial role in the development of the anterior (ventral) zebrafish hypothalamic zone (which is the homologous region of the mammalian arcuate nucleus) with regard to conveying the identity of melanocortin neurons important for food intake and weight regulation (Nasif et al., 2015).

Since both the intermediate hypothalamic nucleus (IN) and the anterior part of Hc express mainly *TH2* (which is not visualized with TH immunohistochemistry, see above), we cannot evaluate whether the dopaminergic cells in these two regions (see Yamamoto et al., 2011) are dependent on *islet1*, although this seems likely.

The torus lateralis (TLa) as well as the corpus mamillare (CM) are completely free of *islet1*-GFP positive cells, as are the diffuse and central nuclei (DIL, CIL) of the inferior lobe (Figure 6B). Furthermore, the massive fiber masses emerging from the periventricular hypothalamus (Hv, Hd) do also not extend into these more lateral hypothalamic areas of the inferior lobe. Clearly, these lateral hypothalamic areas are not dependent on *islet1* expression.

A conspicuous tract (asterisks in Figure 2) emerges from the fiber masses which originate in Hd/Hv and it is observed to run caudally through the midbrain floor into the rhombencephalon and to terminate in the medial part of the secondary gustatory nucleus (SGN; Figure 4D). Indeed, Morita et al. (1983) have earlier shown in the closely related goldfish that retrogradely labeled cell bodies in Hd are seen after SGN tracer injections. In goldfish, this medial part of the SGN is in turn the origin of gustatory fibers to the inferior lobe (Rink and Wullimann, 1998). Thus, as similarly shown in a tracing study by Yáñez et al. (2016), we conclude to observe in the zebrafish this hypothalamo-secondary gustatory nuclear tract with *islet1*-GFP and that there are reciprocal connections between hypothalamus and SGN.

#### Preoptic Region

In amniotes, much of what we address here as zebrafish preoptic region is part of the alar plate hypothalamus. Recently, the identity of the teleostean magnocellular preoptic nucleus (PM) was discussed to be part of the so-called supraopto-paraventricular region seen in land vertebrates (SPV; Herget et al., 2014). The SPV is different in gene expression from the basal plate hypothalamus and is, as a result thereof, the home of the paraventricular nucleus, the core nucleus of the stress-axis, and a wealth of neuropeptidergic neurons develop in this vicinity in all vertebrates (Bardet et al., 2008; Moreno et al., 2012; Puelles et al., 2012; Domínguez et al., 2013; Affaticati et al., 2015; Díaz et al., 2015). Thus it would seem that only part of the zebrafish anterior preoptic parvocellular preoptic nucleus (PPa) corresponds to the two small preoptic nuclei seen in mammals while the remaining teleostean “preoptic” nuclei (PM, PPp, and SC) correspond to the most anterior part of the amniote hypothalamus (see discussion in Herget et al., 2014).

In this (alar) preoptic region there is a small, very caudally located *sonic hedgehog* expression domain in the larval zebrafish which presumably acts in a ventralizing fashion (Figure 6A1, A2). This is a necessary developmental antagonist action toward dorsalizing factors in the anterior forebrain because neither floor nor basal plates are present at this anterior level of the neuraxis (Puelles and Rubenstein, 2003). Not surprisingly then, we find also strong *islet1*-GFP expression in the anterior and posterior parvocellular preoptic region (with some additional cells in PM and SC) of the adult zebrafish (Figure 6B). The mentioned SPV region expresses in all vertebrates the transcription factor coding gene *orthopedia* (*otp*) and a series of other genes, but not *islet1* (Herget et al., 2014). In fact, *islet1* and a suite of other different genes characterize the developing surrounding “preoptic” area. This is in line with our adult *islet1*-GFP data because they show PPa and PPp with profuse *islet1*-GFP expression, but only a few cells in the PM and SC. Thus, the restricted population of *islet1*-GFP positive cells in PM must arise from an *otp*-free zone of the preoptic region (unlike the neuropeptidergic cells of the PM).

Another interesting fact is that a very distinct terminal field of *islet1*-GFP fibers is seen to terminate in the intermediate nucleus of the ventral telencephalon (Vi; Figure 3C), the proposed zebrafish homolog of the medial amygdala (Biechl et al., 2017). These fibers seemingly arise from *islet-GFP* positive cells of the preoptic area (likely PPa) and/or of the more posterior *islet1*-GFP positive hypothalamus. Such projections have indeed been reported in the mouse both from the true mammalian preoptic nuclei as well as from the hypothalamus (Cádiz-Moretti et al., 2016). These authors report the strongest input to originate in the magnocellular preoptic nucleus (importantly, this is not the zebrafish PM, but rather comparable to PPa), ventromedial hypothalamic, dorsal and ventral premammillary nuclei, and posterior hypothalamic area, but not from the neuropeptidergic paraventricular nucleus which corresponds to the zebrafish PM (Herget et al., 2014) and which remains mostly *islet1*-GFP free (see above).

#### Telencephalon

Finally, we see strong *islet1*-GFP expression in part of the subpallium (ventral telencephalon), in contrast to the dorsal telencephalon (pallium and olfactory bulb), which remains completely free of *islet1-GFP* expression (Figure 6B). This expression pattern might be the consequence of *sonic hedgehog* signaling from the preoptic region since a separate basal telencephalic *shh* expression as seen in amniotes has not been described in embryonic zebrafish (Ekker et al., 1995; Strähle et al., 1996; Holzschuh et al., 2003). Alternatively, a later emerging basal telencephalic *shh* expression spot might be responsible for inducing the subpallial *islet1* expression. Clearly, the signaling pathway acting in telencephalic *islet1* induction needs investigation.

An interesting fact regarding the subpallium is that *islet1*-GFP is seen in the ventral division of the dorsal nucleus of the ventral telencephalon (Vdv) – in addition to expression in the entire ventral nucleus (Vv). This is in line with our previous finding that the dorsal nucleus of the ventral telencephalon is divided into pallidal (Vdv) and striatal (Vdd) domains as demonstrated by diagnostic differential gene expression of the LIM/homeodomain genes *lhx6* and *lhx7* (Mueller et al., 2008). These two genes are both only expressed in the ventral division (Vdv) and absent in the dorsal division (Vdd) of the dorsal nucleus, similar to the expression of *islet1* observed here. Moreover, we see populations of *islet1*-GFP cells in the supracommissural nucleus of the ventral telencephalon (Vs), but again only in the ventral part, indicating a subdivision also in Vs as similarly seen in Vd. Thus, we agree with Ganz et al. (2012) that *islet1* is only expressed partially in the subpallium, namely within Vv and part of Vs and not in the central (Vc) and lateral nucleus (Vl), but disagree with the interpretation that the subpallial *islet1* domain is newly wholly defined as Vv and that only the *islet1* negative dorsal subpallial domain (our Vdd) is Vd (see also discussion above). Our previous and current suggestion that Vd represents the basal ganglia, including pallidum (Vdv) and striatum (Vdd), is not only historically founded earlier and has thus priority, it also matches the comparative and functional context reasonably. Furthermore, no *islet1*-GFP cell bodies are seen in the intermediate nucleus of the ventral telencephalon (Vi; Figure 3C) which is the proposed homolog of the medial amygdala (Biechl et al., 2017).

### Further Analysis Using TH

As a further means of corroborating particular neuroanatomical identifications we visualized immunohistochemically tyrosine hydroxylase (TH), the rate-limiting enzyme for catecholamines. This allows for the recognition of dopaminergic and noradrenergic systems along the zebrafish brain neuraxis. Notably, all forebrain TH populations are dopaminergic because of a lack of dopamine-β-hydroxylase there, which is only used in noradrenaline/adrenaline production in hindbrain TH populations (Tillet and Thibault, 1989; Ma, 1994a,b, 2003; Smeets and Reiner, 1994a,b). Telencephalic dopamine cells (incl. olfactory bulb) never colocalize with *islet1*-GFP despite massive *islet*1-GFP expression there, but dopaminergic anterior and posterior parvocellular preoptic cells (PPa, PPp) do colocalize with *islet1*-GFP. In contrast, the dopamine cells seen in magnocellular preoptic nucleus (PM) do not colocalize with *islet1*-GFP. This is in line with the fact that SVP cells derive from *otp*-expressing progenitors (see above) which do not express *islet1* (Herget et al., 2014). The few *islet1*-GFP cells in PM (see section “Results”) must therefore originate from another embryonic source outside of the SVP and become located in the PM as it is defined morphologically. Also, the TH cells in the suprachiasmatic nucleus do not colocalize with *islet1*-GFP.

In the basal plate diencephalon where also *islet1*-GFP expression is seen, only small dopaminergic neurons of the periventricular posterior tuberculum (TPp-p) colocalize with *islet1*-GFP, but never the large dopaminergic pear-shaped projection neurons (TPp-m) and also not the dopamine cells of the paraventricular organ (PVO) and the posterior tuberal nucleus (PTN) where *islet1*-GFP cells are generally absent. The anterior part of the caudal periventricular zone of the hypothalamus contains very many *islet1*-GFP cells, which is in contrast to its caudal part surrounding the posterior ventricular recess. The dopamine cells in the latter, thus, do not colocalize with *islet1-*GFP. The intermediate hypothalamic nucleus (IN), like the anterior part of Hc, both contain *islet1-*GFP cells, but we cannot tell whether these co-localize with dopamine cells because the intermediate nucleus and the anterior Hc uses *TH2* (invisible to immunostaining) for dopamine synthesis (see section “Discussion” above). Thus, within the forebrain basal plate (periventricular posterior tuberculum and hypothalamus) only the TPp-p contains dopaminergic populations colocalizing with *islet1*-GFP and can, thus, be considered to be under the ventralizing influence of the *shh-islet1* signaling pathway, although the same might also apply to the dopamine cells of IN and anterior part of Hc (see section “Discussion” above), but definitely does not apply to those of the remaining posterior tubercular systems (TPp-m, PVO, and PTN).

Regarding the diencephalic alar plate, the ventral thalamic dopamine cells (zona incerta homolog; Wullimann and Rink, 2001; Filippi et al., 2012), but not those of the large dopaminergic pretectal population, colocalize with *islet1-*GFP, in line with the former’s close spatial relationship to the zona limitans intrathalamica (see section “Discussion” above).

Finally, there is also no colocolization of catecholaminergic (mostly noradrenergic) locus coeruleus or area postrema neurons with *islet1*-GFP cell bodies which is in line with their alar plate origin. However, some colocalizing catecholamine neurons are seen close to the vagal motor nucleus (the vagal noradrenergic population, Ma, 1994a,b, 1997; Kress and Wullimann, 2012).

## Conclusion

There are some general conclusions to be drawn from this study. In the adult zebrafish brain (3 and 6 months), *islet1* is apparently still expressed in the same structures as in the late larva. An exception is the absence of positivity in cranial nerve ganglia.

With due caution we conclude that in the 3 months adult zebrafish brain – as a rule – various basal plate populations express *islet1* as a consequence of ventral longitudinal *sonic hedgehog* expression in the floor (midbrain/hindbrain) and basal plates (posterior diencephalon, hypothalamus). These systems are mostly cholinergic motor neurons of the mid- and hindbrain, but include some other systems there as well (e.g., superior reticular formation and rostral perilemniscal nucleus, as well as spino-occipital cells). In the basal plate forebrain, the *islet1* positive systems are the nucleus of the medial longitudinal fascicle (Nmlf), the parvocellular part of the periventricular posterior tubercular nucleus (TPp-p), the ventral, dorsal and anterior part of caudal periventricular hypothalamic zones (Hv, Hd, and Hc), including the intermediate hypothalamic nucleus (IN; Figure 6B).

Although sonic hedgehog signaling is an important upstream factor in brain and spinal cord ventralization (see section “Introduction”), additional locally different signaling molecules and networks of transcription factors are acting along the anteroposterior axis of the CNS and *islet1* expression is only one of many effects of ventralization. However, two exceptional cases of zebrafish forebrain alar plate *islet1* expression may have developmentally plausible explanations. The expression in the alar plate dorsal and ventral thalamus (Figure 6B) might arise from influences through early *sonic hedgehog* signaling from the transversely (not longitudinally, as usual for *shh* expression) positioned zona limitans intrathalamica. Interestingly, some posterior tubercular systems (TPp-m, PVO, and PTN) do not express *islet1* (Figure 6B). They may derive from *shh* expressing cells themselves which are extensively present in the posterior tuberculum in larvae (Figure 6A). The alar plate *islet1* expression domains in the telencencephalic subpallium (Vv, Vdv, and Vs) and in the preoptic area (PPa, PM, PPp, and SC) likely arise from influences of *sonic hedgehog* expression in the embryonic/larval preoptic region (see Figure 6B) or a possibly later emerging *shh* expression domain in the basal telencephalon.

The only catecholamine systems that colocalize with *islet1* are the anterior and posterior periventricular preoptic nuclei (PPa and PPp), the parvocellular periventricular posterior tubercular nucleus (TPp-p), the ventral thalamus (zona incerta homolog), and some cells in the vagal group. The cholinergic system colocalizing with *islet1* are all motornuclei (including the Edinger–Westphal nucleus), the efferent octavolateralis neurons and a few cholinergic cells ventrolateral to the cholinergic caudal perilemniscal nucleus.

## Author Contributions

SB and DB created the immunohistological material. SB, DB and MW contributed to the analysis of the data, created the figures and tables, and wrote the manuscript.

## Conflict of Interest Statement

The authors declare that the research was conducted in the absence of any commercial or financial relationships that could be construed as a potential conflict of interest.
